# The metabolic profile of *Bifidobacterium dentium* reflects its status as a human gut commensal

**DOI:** 10.1186/s12866-021-02166-6

**Published:** 2021-05-24

**Authors:** Melinda A. Engevik, Heather A. Danhof, Anne Hall, Kristen A. Engevik, Thomas D. Horvath, Sigmund J. Haidacher, Kathleen M. Hoch, Bradley T. Endres, Meghna Bajaj, Kevin W. Garey, Robert A. Britton, Jennifer K. Spinler, Anthony M. Haag, James Versalovic

**Affiliations:** 1 Department of Pathology and Immunology, Baylor College of Medicine, Houston, TX USA; 2 Department of Pathology, Texas Children’s Hospital, Houston, TX USA; 3 Department of Regernative Medicine & Cell Biology, Medical University of South Carolina, SC Charleston, USA; 4 Department of Virology and Microbiology, Baylor College of Medicine, Houston, TX USA; 5 Department of Pharmacy Practice and Translational Research, University of Houston College of Pharmacy, Houston, TX USA; 6 Department of Chemistry and Physics, and Department of Biotechnology, Alcorn State University, Lorman, MS 39096 USA

**Keywords:** Bifidobacteria, Metabolism, Carbohydrates, Glycans, Acid stress, Intestine, Commensal

## Abstract

**Background:**

Bifidobacteria are commensal microbes of the mammalian gastrointestinal tract. In this study, we aimed to identify the intestinal colonization mechanisms and key metabolic pathways implemented by *Bifidobacterium dentium*.

**Results:**

*B. dentium* displayed acid resistance, with high viability over a pH range from 4 to 7; findings that correlated to the expression of Na+/H+ antiporters within the *B. dentium* genome. *B. dentium* was found to adhere to human MUC2+ mucus and harbor mucin-binding proteins. Using microbial phenotyping microarrays and fully-defined media, we demonstrated that in the absence of glucose, *B. dentium* could metabolize a variety of nutrient sources. Many of these nutrient sources were plant-based, suggesting that *B. dentium* can consume dietary substances. In contrast to other bifidobacteria, *B. dentium* was largely unable to grow on compounds found in human mucus; a finding that was supported by its glycosyl hydrolase (GH) profile. Of the proteins identified in *B. dentium* by proteomic analysis, a large cohort of proteins were associated with diverse metabolic pathways, indicating metabolic plasticity which supports colonization of the dynamic gastrointestinal environment.

**Conclusions:**

Taken together, we conclude that *B. dentium* is well adapted for commensalism in the gastrointestinal tract.

**Supplementary Information:**

The online version contains supplementary material available at 10.1186/s12866-021-02166-6.

## Introduction

Bifidobacteria are important members of the Actinobacteria phylum within the human intestinal microbiota [1–10]. The establishment of bifidobacteria in the intestine is connected with beneficial health effects, including immune development, neuromodulation, inhibition of pathogens, and modulation of the intestinal microbiota composition [11–23]. To produce these beneficial effects, bifidobacteria must be able to survive gastrointestinal (GI) transit and persist in the dynamic environment of the intestine. Thus, analysis of the mechanisms of intestinal survival and colonization are pivotal to understand the functional activities of bifidobacteria.

Nutrient availability and utilization shapes the composition and gene expression of the intestinal microbiota [11, 24–30]. Broad genomic approaches have predicted that bifidobacteria can use a wide variety of nutrient sources to colonize the human GI tract [5, 25, 31–35]. More direct studies that have examined growth parameters of bifidobacteria have largely focused on carbohydrate metabolism [36]. As a result, information about the physiology and metabolic profiles of any one *Bifidobacterium* species is fragmented. Identifying the strategies used by specific bifidobacteria to harvest dietary nutrients is important for defining the metabolic properties that underpin ecological fitness in and adaptation to the human intestinal environment. Moreover, this information could be employed to increase the presence of select bifidobacteria in the intestine and harness their associated health benefits.

The aim of this study was to identify key pathways in ecological niche development of *Bifidobacterium dentium*. *B. dentium* is a member of the oral and intestinal microbiome. It is frequently isolated from healthy infant stool [3, 6–8] and has an approximate relative abundance of 0.7% in healthy human adults according to the Human Microbiome Project consortium [37–41]. We have previously demonstrated that *B. dentium* colonizes gnotobiotic mice, promotes goblet cell maturation, secretion of the mucin protein MUC2, stimulates intestinal serotonin production, generates the neurotransmitter γ-aminobutyric acid (GABA), alleviates visceral hypersensitivity and regulates the gut-brain-axis [21–23, 38, 42]. The importance of these functions in GI health motivated us to characterize the metabolic profile of *B. dentium* to identify environmental queues that can influence intestinal colonization.

We sought to characterize the metabolic capacity of *B. dentium* using microbial phenotype microarray technology, genome analysis and proteomics. This work is among the first to delineate the metabolic profile of *B. dentium* ATCC 27678. Our data suggest that *B. dentium* adheres to the intestinal mucus layer, exhibits acid resistance, and utilizes a wide range of physiologically abundant dietary nutrient sources commonly found in the intestine. These data suggest that *B. dentium* is well-adapted for life in the gastrointestinal tract.

## Methods

### Bacterial culture conditions

*Bifidobacterium dentium* ATCC 27678 (ATCC, American Type Culture Collection) was grown in de Man, Rogosa and Sharpe (MRS) medium (Difco) in an anaerobic workstation (Anaerobe Systems AS-580) at 37 °C overnight in a mixture of 5% CO_2_, 5% H_2_, and 90% N_2._ Bacterial growth was measured by optical density (OD_600nm_) using a spectrophotometer. For intestinal adhesion assays, *B. dentium* was grown overnight in MRS anaerobically at 37 °C and bacterial cells were pelleted by centrifugation at 5000 x *g* for 5 min. Cell pellets were washed three times with sterile anaerobic PBS to remove residual MRS and the bacterial pellet was resuspended in anaerobic PBS containing 10 μM carboxyfluorescein diacetate succinimidyl ester (CFDA-SE; Thermo Fisher Scientific, Waltham, MA; #V12883) and incubated for 1 h anaerobically at 37 °C. Following incubation, bacterial cells were pelleted by centrifugation at 5000 x *g* for 5 min, and were washed 3-5x with sterile anaerobic PBS. *B. dentium* fluorescence was confirmed by microscopy and were used for adhesion with the HT29-MTX mammalian cell cultures.

For an acid stress test, *B. dentium* was grown in MRS anaerobically at 37 °C for 8 h to exponential phase and bacterial cells were pelleted by centrifugation at 5000 x *g* for 5 min. *B. dentium* was resuspended at an OD_600nm_ = 2.0 in MRS at a pH of 7.6, 7.0, 6.0, 5.0, 4.0, and 3.0 to simulate the different regions of the GI tract. *B. dentium* was incubated anaerobically at 37 °C for 2 h in the various pH conditions. Following incubation, *B. dentium* cells were pelleted by centrifugation at 5000 x *g* for 5 min, washed 2x to remove residual MRS and then resuspended in anaerobic PBS. Cells were stained with the LIVE/DEAD BacLight Bacterial Viability Stains (Thermo Fisher Scientific cat# L7012) according to the manufacturer’s details. Briefly, *B. dentium* was mixed with a 2x LIVE/DEAD BacLight staining reagent mixture and incubated for 15 min in the dark at 37 °C anaerobically. Then a 100 μL volume of each of the *B. dentium* cell suspensions were added to a black-walled 96-well flat-bottom microplate. Fluorescence was recorded using the following excitation (ex) and emission (em) wavelengths: ex: 485 nm/em: 530 nm (green) and ex: 485 nm/em: 630 nm (red) on a Synergy H1 Microplate Reader (Bio-Tek Instruments, Inc.). Viabilities were calculated with the following equation: (ex: 485/em: 530 values)/(ex:458/em: 630 values) × 100% (Ratio green/red × 100%).

### Intracellular pH assay

*B. dentium* was grown in MRS for 24 h from a starter culture inoculated at OD_600nm_ = 0.1. From this starter culture, a 100 μL volume of bacterial suspension was transferred to a conical bottomed 96-well plate and pelleted by centrifugation at 2000 x *g* for 5 min. Cell pellets were washed twice in live cell imaging solution (LCIS, Molecular Probes) and then resuspended in LCIS containing 1x pHrodo Red AM dye (provided as 1000x in dimethyl sulfoxide, DMSO) and 1x PowerLoad (provided as 100x) (Molecular Probes). *B. dentium* was incubated anaerobically at 37 °C for 30 min. Following incubation, bacterial cells were pelleted by centrifugation at 2000 x *g* for 5 min to remove excess staining solution and then were resuspended in a 100 μL volume of LCIS. Using a vacuum manifold with ~ 5 in Hg vacuum pressure, *B. dentium* cells were immobilized on a 0.22 μm-pore polyvinylidene fluoride (PVDF) filter plate (Millipore Sigma, Burlington, MA). Filters were washed once by vacuum and wells were refilled with LCIS. The filter plate was then loaded into a Synergy HT plate reader with incubation at 37 °C. A citrate buffer series was used to examine intracellular pH due to the wide pH range and its successful application with other lactic acid bacteria [43]. Fluorescence (ex: 560 nm/em: 590 nm) was recorded every 5 min over a 50 min timeframe, first in a common buffer (pH 7.6, min 0–10), then in the test buffers at pH 3–8 (min 10–50). Higher relative fluorescence unit (RFU) values indicate more acidic conditions. Standard curves were generated from fluorescence readings taken over 10 min in potassium citrate buffers at pH 4.5, 5.5, 6.5, and 7.5 in the presence of 10 μM valinomycin and 10 μM nigericin to equilibrate intra- and extracellular pH. Intracellular pH was calculated at the final test buffer time point (t = 50 min) from linear regression lines.

### Biolog phenotypic microarray

For Biolog assays, *B. dentium* was grown overnight (~ 16 h) in MRS as described above. Cells were then diluted 1:20 in a fully-defined medium, termed LDM4 (Lactic Acid Bacteria Defined Media 4) [44], lacking glucose. Each well of Biolog NPGM2 and PM1 microarrays (Biolog, Inc., Haywood, CA, USA) was seeded with a 100 μL volume of cell suspension. Growth was monitored by Optical density (OD_600nm_) readings at 10 min intervals for 16 h. Growth was assessed compared to a negative control well lacking any carbon substrate and a value of OD_600nm_ ≥ 0.2 was considered positive (*n* = 2 independent biological replicates per plate).

### Bacterial genome analysis

The genome of *B. dentium* ATCC 27678 (GCF_000172135.1) was downloaded from NCBI and functionally assessed using the web-based tools NCBI Conserved Domain Database, Carbohydrate Active Enzymes (CAZy; www.cazy.org), and KEGG [45–48].

### Mammalian culture conditions

HT29-MTX cells were obtained from Millipore-Sigma (#12040401). Cells were maintained in Gibco Dulbecco’s Modified Eagle Medium (Thermo Fisher Scientific) containing 10% fetal bovine serum (FBS) in a humidified atmosphere at 37 °C, 5% CO_2._ Cells were tested for *Mycoplasma* using the Mycoplasma Detection Kit (Lonza, cat# LT07–518). For adhesion assays, HT29-MTX cells were seeded at 2 × 10^5^ cells on poly-L-lysine coated round coverslips and incubated for 3–5 days until confluent. When monolayers were confluent, HT29-MTX cells were incubated with Hoechst 33342 staining dye solution (Invitrogen) in PBS for 10 min at 37 °C, washed, and treated with 1 × 10^7^ cells of CFDA-tagged *B. dentium* for 1 h aerobically at 37 °C. After the incubation, non-adhered cells were removed with 3x washes of PBS and cells were fixed with Clarke’s Fixative to maintain the mucus architecture. A subset of cells were used for Scanning Electron Microscopy (SEM) imaging using a FEI XL-30FEG microscope. Cells that were reserved for immunostaining were permeabilized with 0.1% Triton-X for 30 min at room temperature, blocked with PBS containing 10% donkey serum, and incubated with an anti-human MUC2 antibody (Santa Cruz, cat # sc-515,032; 1:200 dilution) overnight at 4 °C. Following PBS washes, cells were incubated with donkey-anti-mouse AlexaFluor 555 (Life Technologies, cat # A11004; 1:1000 dilution) for 1 h at room temperature. Coverslips were mounted to slides using FluoroMount (Thermo Fisher Scientific) and slides were imaged on the Nikon Eclipse TiE inverted microscope.

### Scanning electron microscopy (SEM)

Following imaging, the wells of the slides were washed gently with PBS containing Mg^2+^ and Ca^2+^ (2x) and fixed in 2.5% glutaraldehyde in PBS for 1 h at room temperature as previously described [42]. The black compartment of the CELLview slide was detached, the slide was dehydrated with ethanol, and coated in 20 nm of gold using a desktop sputtering system (Denton Desk II). All slides were viewed in a FEI XL-30FEG SEM microscope operated with an electron beam acceleration voltage of 12 kV [42].

### Proteomic analysis

#### Chemical and reagents

Optima LC/MS-grade acetonitrile (ACN), formic acid (FA), and water, and Promega™ porcine trypsin protease were all purchased from Thermo Fisher Scientific. Ammonium bicarbonate (BioUltra-grade) was purchased from Millipore-Sigma.

#### Proteomics sample preparation

Bacterial sample pellets were suspended in a 200-μL volume of water and samples were sonicated in an ultrasonic bath for 30 min. Afterwards, the samples were centrifuged for 5 min at 10,000 rpm. The resulting sample supernatants containing bacterial protein were removed from the pellet of cellular debris, and the samples were dried in a SpeedVac overnight to yield pelleted protein in the sample tubes. A 100-μL volume of a 10 μg/mL solution of porcine trypsin prepared in a 25 mM ammonium bicarbonate solution was added to the pelleted protein contained in each sample tube, and the samples were vortex-mixed for 1 min and incubated at 37 °C for 8 h.

#### Chromatography

Tryptic digest samples were chromatographically separated on a Dionex Ultimate 3000 RSLC nano-system (Thermo Scientific) using an Acclaim PepmapTM C-18 capillary column (75 μm (ID) × 150 mm (L), Thermo Scientific) outfitted with an Acclaim PepmapTM C18 trap column (100 μm (ID) × 20 mm (L), Thermo Scientific). Chromatography was performed as previously described [22]. Elution gradients were prepared from an aqueous mobile phase (A) of H2O:ACN:FA (94.9:5:0.1 *v/v/v*) and an organic mobile phase (B) of ACN:FA (99.9:0.1 v/v). Sample elution onto the trap column was carried out using a trap column buffer of H2O:ACN:FA (94.9:5:0.1 *v/v/v*). Samples (5 μL) were injected onto the trap column with a flow rate of 5 μL/min. After 5 min, the loading valve was switched to allow the sample to elute off the trap column at a flow rate of 300 nL/min and onto the capillary column for separation. The elution gradient used was specified as follows: Started at 1% B, ramped up linearly to 45% B over 37 min; ramped up linearly to 80% B over 1 min; held at 80% B for 1 min; ramped back to 1% B over 1 min and held for 16 min to re-equilibrate.

#### Mass spectrometric conditions

Samples were analyzed using an Orbitrap Fusion mass spectrometer (Thermo Scientific) using a nanoionization source operated in positive ion mode with the following source conditions: ionspray voltage, static at 1.6 kV; ion transfer tube temperature, 275 °C. Global MS acquisition parameters were specified as follows: precursor ion scan range, mass-to-charge (*m/z)* 200 - *m/z* 1000; S-lens RF level, 60%; data type, profile; MIPS, true; charge states, 2–4; data dependent mode, top speed; precursor priority, most intense; exclude after n times, 1: exclusion duration, 60s; mass tolerance, parts-per-million (ppm); low/high, 10; exclude isotopes, true; MSn level, 2; isolation mode, quadrupole; isolation window, *m/z* 1.6; CID activation, true; CID collision energy, 35%; detector type, Orbitrap; scan-range mode, auto; orbitrap resolution, 120,000; automatic gain control (AGC) target, 5.0e4; maximum injection time, 60 ms; microscans, 1; and, tandem MS data format, profile. Data were acquired with the Thermo Scientific Xcalibur software package (v4.1.50).

#### Mass spectrometric data analysis

Data were analyzed using Proteome Discoverer (Thermo Scientific). Data were searched against the Uniprot *Bifidobacterium* database (8 Aug 2020) which also included a common contaminant database. The following parameters were used for protein identification: minimum precursor mass, 350 Dalton (Da); maximum precursor mass, 5000 Da; minimum peak count, 1; minimum peptide length, 6; precursor mass tolerance, 10 ppm; fragment mass tolerance, 0.02 Da; dynamic modifications included oxidation for methionine and acetylation for protein N-terminus; target and decoy database, concatenated; validation based on q-Value; and, FDR targets were 0.01 for strict and 0.05 for relaxed.

### Statistics and graphs

Graphs and heat maps were created using GraphPad Prism software (version 8) (GraphPad Inc.). Comparisons were made with either One-way ANOVA or Repeated Measures ANOVA with the Holm-Sidak *post-hoc* test. The data are presented as mean ± standard deviation, with *P* < 0.05 (*) considered statistically significant. See Tables 3 and 4 and Supplemental Table 1 and Supplemental Table 2 for statistical analysis.

## Results

### *B. dentium* is acid resistant and can adhere to intestinal mucus suggesting its efficacy to persist in the gastrointestinal tract

**Fig. 1:**
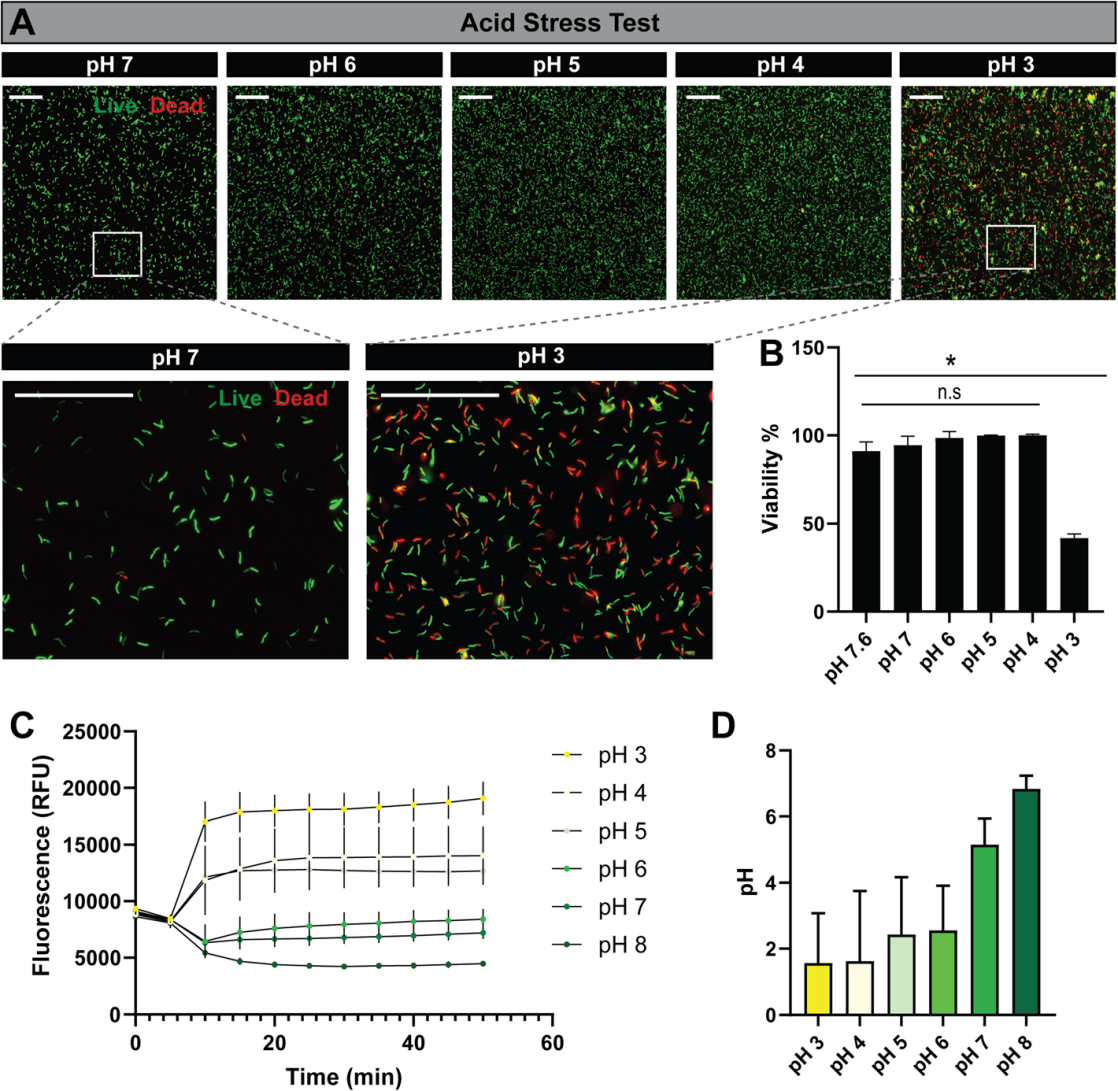
*Bifidobacterium dentium* is resistant to acid stress. **a** Representative images of live/dead staining of *B. dentium* ATCC 27678 after 2 h incubation in media at pH 7, 6, 5, 4, and 3. Inserts at high magnification highlight the abundance of live (green) and dead (red) *B. dentium* at pH 7 and pH 3 (scale bar = 50 μm). **b** Quantitation of live/dead cell staining on a fluorescent plate reader. **c** Intracellular pH analysis of *B. dentium* with pHrodo Red pH sensitive dye. Variance in intracellular pH is reflected by the change in relative fluorescence units (RFU), at various extracellular pH values over 50 min. **d** Calculated final intracellular pH values at t = 50 min. All data are presented as mean ± stdev

To colonize the gastrointestinal tract microbes must overcome the acidic pH found in the stomach and upper GI to gain access to the lower parts of the intestine. In general, bifidobacteria are considered to have a weak acid tolerance with the exception of *B. animalis* [49] and *B. longum* [50–52]. Using the NCBI Conserved Domain Database to assess the functional annotation of the *B. dentium* ATCC 27678 (GCF_000172135.1) proteins, we noted the presence of three Na^+^/H^+^ antiporter proteins that may contribute to acid tolerance in *B. dentium* [45–48] (Table 1). To address the ability of *B. dentium* to survive transit through the low pH environment of the stomach and small intestine experimentally, we incubated overnight cultures of *B. dentium* in MRS with pH of 3, 4, 5, 6, and 7 for 2 h. After incubation, cell viability was obtained by live/dead cell staining using a BACLight kit as examined by microscopy (Fig. 1a) and fluorescence plate reader quantification (Fig. 1b). *B. dentium* exhibited high viability over a pH range from 4 to 7, as denoted by green staining, and > 90% viability levels. Even in highly acidic conditions (pH 3), *B. dentium* still maintained 41.8% ± 2.4 viability, indicating acid tolerance. Intracellular pH analysis by pHrodo Red AM dye demonstrated that surviving *B. dentium* were able to regulate their intracellular pH over time (Fig. 1c, d). These data suggest that *B. dentium* is acid-tolerant, similar to findings with gastrointestinal colonizers *B. animalis* and *B. longum* [53], and thus likely able to survive the transit through the upper GI system.

**Table 1:** Notable ion antiporters identified from the genome *of Bifidobacterium dentium* ATCC 27678

Accession No.	Description	Proposed Function
WP_003840740.1	Na^+^/H^+^ antiporter	Acid tolerance
WP_003837813.1	cation:proton antiporter	Acid tolerance
WP_003838459.1	Na^+^/H^+^ antiporter	Acid tolerance

**Fig. 2:**
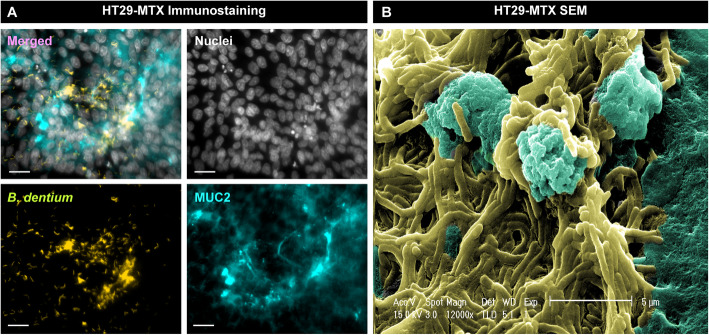
*B. dentium* adheres to mucus-producing human intestinal epithelial cells. **a** Representative immunofluorescence images of *B. dentium* ATCC 27678 (yellow) co-localization with MUC2 (blue) in mucin-producing human HT29-MTX colonic cells after 1 h incubation (scale bar = 50 μm). **b** Scanning electron micrograph of *B. dentium* and HT29-MX cells after 1 h incubation (scale bar = 5 μm)

The ability to adhere to the intestinal mucus layer is an important aspect of bifidobacterial colonization [54]. Mucus adhesion is proposed to enhance epithelial integrity and pathogen exclusion [55], as well as provide closer access for metabolite delivery and immune stimulation [56, 57]. Investigation of the functional annotation of *B. dentium* ATCC 27678 indicated the presence of glycosyltransferase enzymes that promote bacterial capsular formation along with pilin and fimbrial proteins (Table 2). These proteins have been previously associated with mucus adherence and GI colonization and may also facilitate mucus adhesion for *B. dentium*. To assess adhesion of *B. dentium* to intestinal mucus, we added fluorescently-tagged *B. dentium* to human mucin-producing HT29-MTX monolayers for 1 h and examined adhesion by immunostaining (Fig. 2). Similar to other well characterized *Bifidobacterium* strains, we observed robust adhesion of *B. dentium* to MUC2 mucin by immunostaining (Fig. 2a) and SEM imaging (Fig. 2b). The ability of *B. dentium* to withstand acidic conditions and adhere to intestinal mucus highlights its potential to inhabit the intestine.

**Table 2:** Notable glycosyltransferases and proteins involved in adhesion identified from the genome *of Bifidobacterium dentium* ATCC 27678

Accession No.	Description	Proposed Function
WP_003837192.1	Glycosyltransferase family 2	Bacterial capsule biosynthesis
WP_003837196.1	Glycosyltransferase family 2	Bacterial capsule biosynthesis
WP_003836797.1	Glycosyltransferase family 2	Bacterial capsule biosynthesis
WP_003836799.1	Glycosyltransferase family 1	Exopolysaccharide biosynthesis
WP_003837542.1	Glycosyltransferase family A	mannose-1-phosphate guanylyltransferase
WP_003837819.1	Glycosyltransferase family 2	Bacterial capsule biosynthesis
WP_003838069.1	Glycosyltransferase family B	GT transferase
WP_034257238.1	Glycosyltransferase family 4 protein	Cell wall biosynthesis
WP_033488900.1	Glycosyltransferase	Anthranilate phosphoribosyltransferase
WP_034257219.1	Nucleotidyltransferases	2-C-methyl-D-erythritol 4-phosphate cytidylyltransferase
WP_003837207.1	Isopeptide-forming fimbrial protein	Pilus formation

### *B. dentium* metabolism of dietary sugars and select host derived carbon sources promote growth

**Fig. 3:**
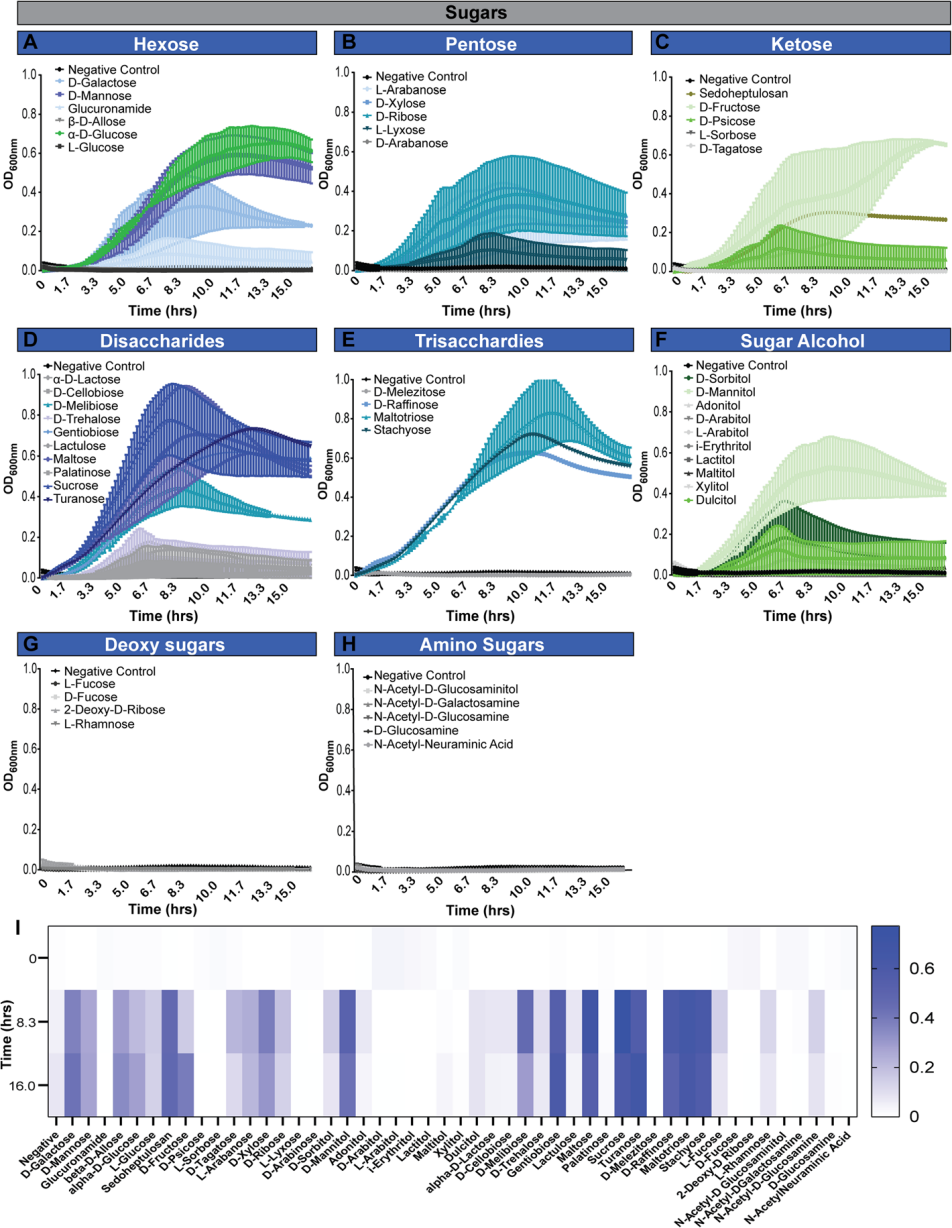
*B. dentium* grows on select sugars in the absence of glucose. *B. dentium* ATCC 27678 was grown anaerobically at 37 °C in Biolog plates with a fully-defined media (LDM4) preparation that lacked glucose. Growth was monitored over 16 h by plate reader in plate containing **a** hexoses, **b** pentoses, **c** ketoses, **d** disaccharides, **e** trisaccharides, **f** sugar alcohols, **g** deoxy sugars and **h** amino sugars. **i** For visualization, heat maps were generated for all sugars at time 0, 8.3 and 16.0 h. All data are presented as mean ± stdev

Previous work examining microbe metabolism have relied on adding nutritional components to rich media, such as MRS. However, the complexity of this medium, poses challenges to identification of the dietary requirements of these microbes. To circumvent this challenge we used LDM4 media, a fully-defined medium which can be prepared to exact nutrient composition [44]. Using LDM4 prepared without glucose, we examined the ability of *B. dentium* to grow on a number of nutrients as individual primary carbon sources by Biolog phenotype analysis (Fig. 3, Tables 3 and 4). Growth of *B. dentium* was examined in the presence of 50 different sugars, including hexoses (Fig. 3a), pentoses (b), ketoses (c), disaccharides (d), trisaccharides (e), sugar alcohols (f), deoxy sugars (g) and amino sugars (h). As shown in the graphs and heat map (Fig. 3i), *B. dentium* exhibits robust growth with a variety of carbohydrates, with substantial growth found on galactose, mannose, maltose, xylose, sucrose, truanose, D-raffinose, maltotriose, stachyose, D-melibiose, gentiobiose, sedoheptulosan and D-mannitol. These findings are consistent with the *B. dentium* Bd1 genome analysis [33], which indicated that *B. dentium* encoded a wide variety of enzymes for the fermentation of pentose sugars. The utilization of sucrose by *B. dentium* ATCC 27678 was reflected by our proteomic analysis, in which we identified 24 proteins involved in sucrose metabolism (Table 5). We also identified proteins involved in maltose-binding (MalE), maltose transport systems (MalG), xylose isomerases, xylose ABC transporters, raffinose-binding, mannose metabolism, and ABC sugar transports (Table 5); findings which reflect our growth profiles. No appreciable growth was observed on many sugars, including D- or L-arabitol, lactitol, maltitol, D-lactose, D-cellboiose, D-trehalose, lactulose, fucose, among others. The inability of *B. dentium* ATCC 27678 to use fucose is consistent a previous study that demonstrate that *B. dentium* DSM 20436 and VBif10D2 are unable to use fucose in mYCFA medium [58]. In this capacity, *B. dentium* resembles most *Bifidobacterium* species which are largely unable to use fucose [58].

**Table 3:** Statistics from growth curves at time point, 8.3 h. Significant *p* values are denoted as follows: *P < 0.05, **P < 0.01, ***P < 0.001, ****P < 0.0001

	8.3 hrs		
**Comparison**	**95.00% CI of diff.**	**Significant?**	***P*** **Value**
Negative vs. D-Galactose	-0.7584 to -0.09959	**	0.0022
Negative vs. D-Mannose	-0.8634 to -0.2046	****	<0.0001
Negative vs. alpha-D-Glucose	-0.8554 to -0.1966	****	<0.0001
Negative vs. Sedoheptulosan	-0.7294 to -0.07059	**	0.0061
Negative vs. D-Xylose	-0.6594 to -0.0005865	*	0.0492
Negative vs. D-Mannitol	-0.7794 to -0.1206	**	0.0011
Negative vs. D-Melibiose	-0.7194 to -0.06059	**	0.0084
Negative vs. Gentiobiose	-0.7994 to -0.1406	***	0.0005
Negative vs. Maltose	-0.9494 to -0.2906	****	<0.0001
Negative vs. Sucrose	-1.049 to -0.3906	****	<0.0001
Negative vs. Turanose	-0.8294 to -0.1706	***	0.0002
Negative vs. D-Raffinose	-0.8694 to -0.2106	****	<0.0001
Negative vs. Maltotriose	-0.9094 to -0.2506	****	<0.0001
Negative vs. Stachyose	-0.8794 to -0.2206	****	<0.0001
Negative vs. D-Gluconic Acid	-0.5014 to -0.03863	**	0.0097
Negative vs. L-Proline	-0.7237 to -0.03635	*	0.0206
Negative vs. Sec-Butylamine	0.004907 to 0.03509	*	0.013
Negative vs. Amygdalin	-0.4010 to -0.09401	****	<0.0001
Negative vs. Arbutin	-0.3325 to -0.02551	*	0.0107
Negative vs. Salicin	-0.3335 to -0.02651	*	0.01
	16 hrs		
**Comparison**	**95.00% CI of diff.**	**Significant?**	***P*** **Value**
Negative vs. D-Mannose	-0.8481 to -0.2299	****	<0.0001
Negative vs. alpha-D-Glucose	-0.9221 to -0.3039	****	<0.0001
Negative vs. Sedoheptulosan	-0.6891 to -0.07092	**	0.0052
Negative vs. D-Mannitol	-0.6491 to -0.03092	*	0.0198
Negative vs. Gentiobiose	-0.8191 to -0.2009	****	<0.0001
Negative vs. Maltose	-0.7591 to -0.1409	***	0.0004
Negative vs. Sucrose	-0.8091 to -0.1909	****	<0.0001
Negative vs. Turanose	-0.8791 to -0.2609	****	<0.0001
Negative vs. D-Raffinose	-0.7291 to -0.1109	**	0.0012
Negative vs. Maltotriose	-0.8391 to -0.2209	****	<0.0001
Negative vs. Stachyose	-0.7891 to -0.1709	***	0.0001
Negative vs. D-Gluconic Acid	-0.3574 to -0.08257	****	<0.0001
Negative vs. Amygdalin	-0.6605 to -0.4385	****	<0.0001
Negative vs. Inosine	-0.2255 to -0.003522	*	0.038
Negative vs. Arbutin	-0.4595 to -0.2375	****	<0.0001
Negative vs. Salicin	-0.4375 to -0.2155	****	<0.0001

**Table 4:** Statistics from growth curves at time point, 16.0 h. Significant p values are denoted as follows: *P < 0.05, **P < 0.01, ***P < 0.001, ****P < 0.0001

Comparison	95.00% CI of diff.	Significant?	***P*** Value
Negative vs. D-Mannose	− 0.8481 to − 0.2299	****	< 0.0001
Negative vs. alpha-D-Glucose	− 0.9221 to − 0.3039	****	< 0.0001
Negative vs. Sedoheptulosan	− 0.6891 to − 0.07092	**	0.0052
Negative vs. D-Mannitol	− 0.6491 to − 0.03092	*	0.0198
Negative vs. Gentiobiose	−0.8191 to − 0.2009	****	< 0.0001
Negative vs. Maltose	−0.7591 to − 0.1409	***	0.0004
Negative vs. Sucrose	−0.8091 to − 0.1909	****	< 0.0001
Negative vs. Turanose	−0.8791 to − 0.2609	****	< 0.0001
Negative vs. D-Raffinose	−0.7291 to − 0.1109	**	0.0012
Negative vs. Maltotriose	−0.8391 to − 0.2209	****	< 0.0001
Negative vs. Stachyose	−0.7891 to − 0.1709	***	0.0001
Negative vs. D-Gluconic Acid	−0.3574 to − 0.08257	****	< 0.0001
Negative vs. Amygdalin	−0.6605 to − 0.4385	****	< 0.0001
Negative vs. Inosine	−0.2255 to − 0.003522	*	0.038
Negative vs. beta-Methyl-DXyloside	−0.06148 to 0.1605	ns	0.9514
Negative vs. Arbutin	−0.4595 to − 0.2375	****	< 0.0001
Negative vs. Salicin	−0.4375 to − 0.2155	****	< 0.0001

**Table 5:** Proteins identified in *B. dentium* ATCC 27678 by proteomic analysis

Pathway Description	Pathway Accession	# Proteins
2-Oxocarboxylic acid metabolism	bde01210	12
2-Oxocarboxylic acid metabolism	bks01210	9
2-Oxocarboxylic acid metabolism	blf01210	5
2-Oxocarboxylic acid metabolism	blx01210	1
2-Oxocarboxylic acid metabolism	bln01210	1
ABC transporters	bde02010	26
ABC transporters	bks02010	6
ABC transporters	blf02010	4
Acarbose and validamycin biosynthesis	bks00525	1
Acarbose and validamycin biosynthesis	bde00525	1
Acarbose and validamycin biosynthesis	boa00525	1
Alanine, aspartate and glutamate metabolism	bde00250	9
Alanine, aspartate and glutamate metabolism	bks00250	4
Alanine, aspartate and glutamate metabolism	blf00250	4
Alanine, aspartate and glutamate metabolism	bln00250	3
Amino sugar and nucleotide sugar metabolism	bde00520	13
Amino sugar and nucleotide sugar metabolism	bks00520	9
Amino sugar and nucleotide sugar metabolism	blf00520	7
Amino sugar and nucleotide sugar metabolism	bln00520	2
Amino sugar and nucleotide sugar metabolism	boa00520	2
Aminoacyl-tRNA biosynthesis	bde00970	19
Aminoacyl-tRNA biosynthesis	bks00970	11
Aminoacyl-tRNA biosynthesis	blf00970	5
Aminoacyl-tRNA biosynthesis	bln00970	4
Aminoacyl-tRNA biosynthesis	boa00970	2
Arginine biosynthesis	bde00220	8
Arginine biosynthesis	bks00220	7
Arginine biosynthesis	blf00220	6
Arginine biosynthesis	bln00220	3
Bacterial secretion system	bde03070	6
Bacterial secretion system	bks03070	3
Bacterial secretion system	blf03070	2
Bacterial secretion system	bln03070	1
beta-Alanine metabolism	bde00410	1
beta-Lactam resistance	bde01501	3
beta-Lactam resistance	blf01501	1
Biosynthesis of amino acids	bde01230	38
Biosynthesis of amino acids	bks01230	23
Biosynthesis of amino acids	blf01230	18
Biosynthesis of amino acids	bln01230	7
Biosynthesis of amino acids	boa01230	4
Biosynthesis of amino acids	blx01230	1
Biosynthesis of antibiotics	bde01130	51
Biosynthesis of antibiotics	bks01130	31
Biosynthesis of antibiotics	blf01130	19
Biosynthesis of antibiotics	bln01130	9
Biosynthesis of antibiotics	boa01130	7
Biosynthesis of antibiotics	blx01130	2
Biosynthesis of antibiotics	blm01130	1
Biosynthesis of secondary metabolites	bde01110	62
Biosynthesis of secondary metabolites	bks01110	37
Biosynthesis of secondary metabolites	blf01110	24
Biosynthesis of secondary metabolites	bln01110	10
Biosynthesis of secondary metabolites	boa01110	8
Biosynthesis of secondary metabolites	blx01110	2
Biosynthesis of secondary metabolites	blm01110	1
Butanoate metabolism	bde00650	4
Butanoate metabolism	bks00650	2
Butanoate metabolism	blf00650	1
Butanoate metabolism	boa00650	1
C5-Branched dibasic acid metabolism	bde00660	4
C5-Branched dibasic acid metabolism	bks00660	3
Carbon metabolism	bde01200	25
Carbon metabolism	bks01200	15
Carbon metabolism	blf01200	10
Carbon metabolism	boa01200	5
Carbon metabolism	bln01200	3
Chloroalkane and chloroalkene degradation	bks00625	1
Chloroalkane and chloroalkene degradation	bde00625	1
Citrate cycle (TCA cycle)	bde00020	5
Citrate cycle (TCA cycle)	bks00020	2
Citrate cycle (TCA cycle)	boa00020	2
Citrate cycle (TCA cycle)	blf00020	1
Cyanoamino acid metabolism	bde00460	1
Cyanoamino acid metabolism	boa00460	1
Cysteine and methionine metabolism	bde00270	8
Cysteine and methionine metabolism	bks00270	6
Cysteine and methionine metabolism	blf00270	3
Cysteine and methionine metabolism	boa00270	2
Cysteine and methionine metabolism	blm00270	1
Degradation of aromatic compounds	bks01220	1
Degradation of aromatic compounds	bde01220	1
DNA replication	bde03030	2
DNA replication	bks03030	1
Fatty acid biosynthesis	boa00061	2
Fatty acid biosynthesis	bks00061	1
Fatty acid biosynthesis	blf00061	1
Fatty acid biosynthesis	bde00061	1
Fatty acid degradation	bks00071	1
Fatty acid degradation	bde00071	1
Fatty acid metabolism	boa01212	2
Fatty acid metabolism	bks01212	1
Fatty acid metabolism	blf01212	1
Fatty acid metabolism	bde01212	1
Fructose and mannose metabolism	bde00051	2
Fructose and mannose metabolism	bks00051	1
Fructose and mannose metabolism	boa00051	1
Galactose metabolism	bde00052	6
Galactose metabolism	bks00052	4
Galactose metabolism	blf00052	3
Galactose metabolism	boa00052	1
Glutathione metabolism	bde00480	3
Glutathione metabolism	bks00480	2
Glutathione metabolism	blf00480	1
Glycerolipid metabolism	bks00561	1
Glycerolipid metabolism	blf00561	1
Glycerolipid metabolism	bde00561	1
Glycerophospholipid metabolism	bde00564	4
Glycerophospholipid metabolism	bks00564	3
Glycerophospholipid metabolism	blf00564	2
Glycerophospholipid metabolism	bln00564	1
Glycine, serine and threonine metabolism	bde00260	9
Glycine, serine and threonine metabolism	bks00260	3
Glycine, serine and threonine metabolism	bln00260	1
Glycine, serine and threonine metabolism	blf00260	1
Glycine, serine and threonine metabolism	boa00260	1
Glycolysis / Gluconeogenesis	bde00010	11
Glycolysis / Gluconeogenesis	bks00010	10
Glycolysis / Gluconeogenesis	blf00010	5
Glycolysis / Gluconeogenesis	bln00010	3
Glycolysis / Gluconeogenesis	boa00010	3
Glycolysis / Gluconeogenesis	blm00010	1
Glyoxylate and dicarboxylate metabolism	bde00630	6
Glyoxylate and dicarboxylate metabolism	bks00630	2
Glyoxylate and dicarboxylate metabolism	blf00630	2
Histidine metabolism	blf00340	1
Histidine metabolism	bde00340	1
Homologous recombination	bde03440	2
Homologous recombination	bks03440	1
Inositol phosphate metabolism	boa00562	2
Inositol phosphate metabolism	bks00562	1
Inositol phosphate metabolism	bde00562	1
Lipopolysaccharide biosynthesis	boa00540	1
Lysine biosynthesis	bde00300	4
Lysine biosynthesis	bks00300	3
Metabolic pathways	bde01100	118
Metabolic pathways	bks01100	71
Metabolic pathways	blf01100	48
Metabolic pathways	boa01100	19
Metabolic pathways	bln01100	17
Metabolic pathways	blx01100	7
Metabolic pathways	blm01100	1
Methane metabolism	bde00680	7
Methane metabolism	bks00680	4
Methane metabolism	bln00680	2
Methane metabolism	blf00680	2
Methane metabolism	boa00680	1
Microbial metabolism in diverse environments	bde01120	36
Microbial metabolism in diverse environments	bks01120	24
Microbial metabolism in diverse environments	blf01120	13
Microbial metabolism in diverse environments	boa01120	6
Microbial metabolism in diverse environments	bln01120	3
Microbial metabolism in diverse environments	blm01120	1
Mismatch repair	bde03430	3
Mismatch repair	bks03430	2
Monobactam biosynthesis	bks00261	2
Monobactam biosynthesis	bde00261	2
Naphthalene degradation	bks00626	1
Naphthalene degradation	bde00626	1
Nicotinate and nicotinamide metabolism	blf00760	2
Nicotinate and nicotinamide metabolism	bks00760	2
Nicotinate and nicotinamide metabolism	bde00760	2
Nitrogen metabolism	bde00910	3
Nitrogen metabolism	bks00910	2
Nitrogen metabolism	blf00910	2
One carbon pool by folate	blf00670	2
One carbon pool by folate	bde00670	2
One carbon pool by folate	bks00670	1
Other glycan degradation	bde00511	1
Oxidative phosphorylation	bde00190	9
Oxidative phosphorylation	bks00190	6
Oxidative phosphorylation	blx00190	5
Oxidative phosphorylation	blf00190	4
Oxidative phosphorylation	bln00190	3
Oxidative phosphorylation	boa00190	2
Pantothenate and CoA biosynthesis	bde00770	6
Pantothenate and CoA biosynthesis	bks00770	4
Pantothenate and CoA biosynthesis	blf00770	3
Pantothenate and CoA biosynthesis	blx00770	1
Pantothenate and CoA biosynthesis	bln00770	1
Pantothenate and CoA biosynthesis	boa00770	1
Pentose and glucuronate interconversions	bde00040	4
Pentose and glucuronate interconversions	blf00040	1
Pentose and glucuronate interconversions	bks00040	1
Pentose phosphate pathway	bde00030	10
Pentose phosphate pathway	bks00030	6
Pentose phosphate pathway	blf00030	5
Peptidoglycan biosynthesis	bde00550	4
Peptidoglycan biosynthesis	bks00550	1
Peptidoglycan biosynthesis	blf00550	1
Phenylalanine, tyrosine and tryptophan biosynthesis	bde00400	2
Phenylalanine, tyrosine and tryptophan biosynthesis	blf00400	1
Phenylalanine, tyrosine and tryptophan biosynthesis	bks00400	1
Phosphotransferase system (PTS)	bks02060	1
Phosphotransferase system (PTS)	bde02060	1
Polyketide sugar unit biosynthesis	bks00523	1
Polyketide sugar unit biosynthesis	bde00523	1
Polyketide sugar unit biosynthesis	boa00523	1
Porphyrin and chlorophyll metabolism	bde00860	1
Propanoate metabolism	bde00640	7
Propanoate metabolism	bks00640	5
Propanoate metabolism	blf00640	3
Propanoate metabolism	blm00640	1
Protein export	bde03060	7
Protein export	bks03060	3
Protein export	blf03060	2
Protein export	bln03060	1
Purine metabolism	bde00230	19
Purine metabolism	bks00230	10
Purine metabolism	blf00230	6
Purine metabolism	bln00230	4
Purine metabolism	blx00230	2
Purine metabolism	boa00230	2
Pyrimidine metabolism	bde00240	11
Pyrimidine metabolism	bks00240	7
Pyrimidine metabolism	blf00240	5
Pyrimidine metabolism	bln00240	3
Pyrimidine metabolism	boa00240	2
Pyrimidine metabolism	blx00240	1
Pyruvate metabolism	bde00620	10
Pyruvate metabolism	bks00620	5
Pyruvate metabolism	boa00620	3
Pyruvate metabolism	blf00620	2
Pyruvate metabolism	blm00620	1
Quorum sensing	bde02024	17
Quorum sensing	bks02024	5
Quorum sensing	blf02024	4
Quorum sensing	bln02024	1
Riboflavin metabolism	boa00740	1
Ribosome	bde03010	47
Ribosome	bks03010	17
Ribosome	bln03010	15
Ribosome	blf03010	14
Ribosome	boa03010	10
Ribosome	blx03010	5
RNA degradation	bde03018	6
RNA degradation	bln03018	4
RNA degradation	bks03018	4
RNA degradation	blf03018	3
RNA degradation	boa03018	2
RNA degradation	bad03018	1
RNA polymerase	bde03020	4
RNA polymerase	bks03020	3
RNA polymerase	blf03020	2
RNA polymerase	blx03020	1
RNA polymerase	bln03020	1
Secondary bile acid biosynthesis	bks00121	1
Secondary bile acid biosynthesis	bde00121	1
Selenocompound metabolism	bde00450	2
Selenocompound metabolism	bks00450	1
Selenocompound metabolism	blf00450	1
Sphingolipid metabolism	bde00600	1
Starch and sucrose metabolism	bde00500	11
Starch and sucrose metabolism	bks00500	6
Starch and sucrose metabolism	blf00500	4
Starch and sucrose metabolism	boa00500	2
Starch and sucrose metabolism	bln00500	1
Streptomycin biosynthesis	bks00521	3
Streptomycin biosynthesis	bde00521	3
Streptomycin biosynthesis	boa00521	2
Streptomycin biosynthesis	blf00521	1
Taurine and hypotaurine metabolism	bde00430	3
Taurine and hypotaurine metabolism	bks00430	2
Taurine and hypotaurine metabolism	blf00430	1
Thiamine metabolism	bde00730	1
Two-component system	bde02020	4
Two-component system	blf02020	2
Two-component system	bks02020	1
Tyrosine metabolism	bks00350	1
Tyrosine metabolism	bde00350	1
Valine, leucine and isoleucine biosynthesis	bde00290	7
Valine, leucine and isoleucine biosynthesis	bks00290	4
Valine, leucine and isoleucine biosynthesis	blf00290	3
Valine, leucine and isoleucine biosynthesis	blx00290	1
Valine, leucine and isoleucine biosynthesis	bln00290	1
Valine, leucine and isoleucine degradation	bde00280	2
Valine, leucine and isoleucine degradation	bks00280	1
Valine, leucine and isoleucine degradation	blf00280	1
Vancomycin resistance	bde01502	1
Vitamin B6 metabolism	bde00750	1

**Fig. 4:**
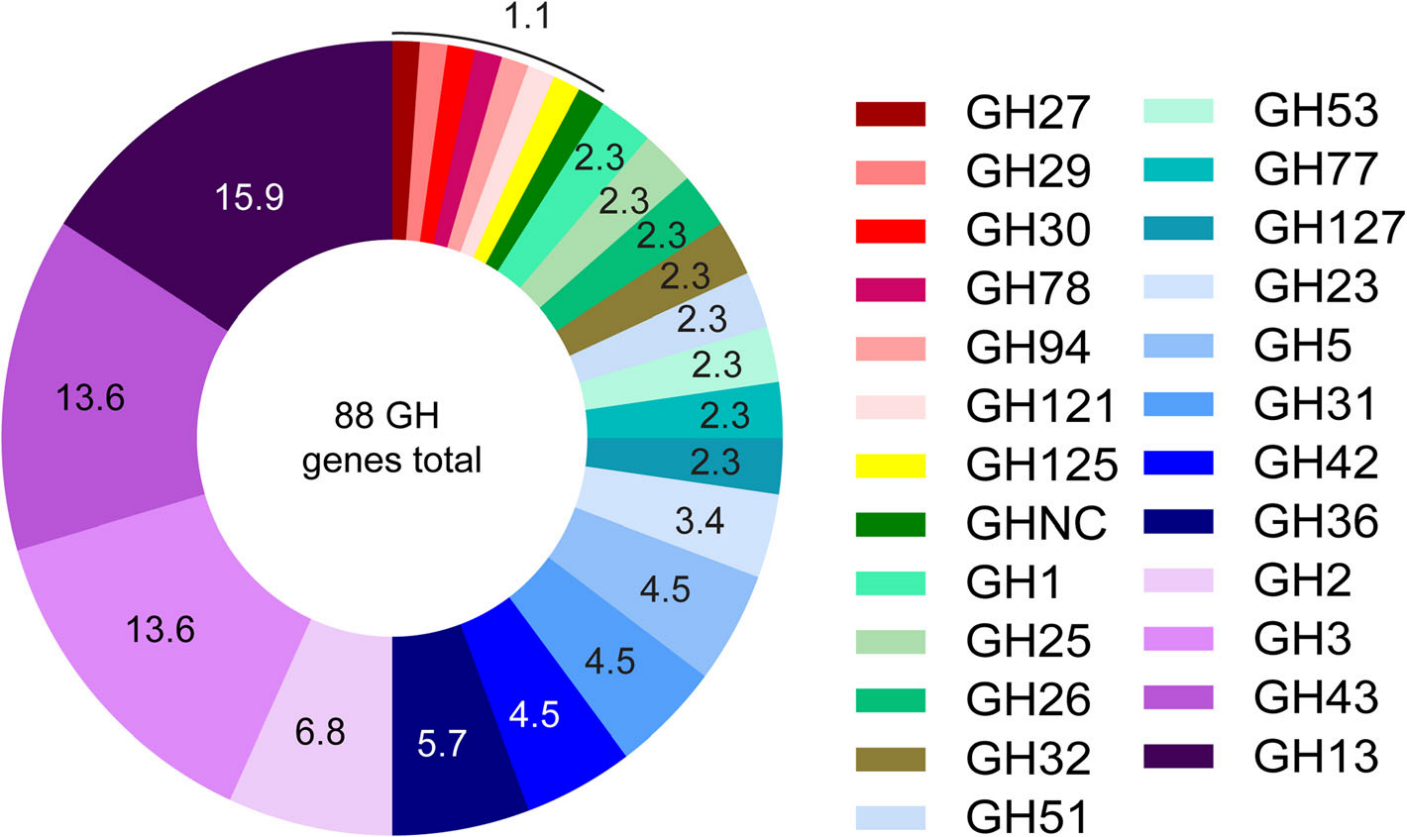
The *B. dentium* ATCC 27678 genome contains mulitple glycosyl hydrolase (GH) genes. The *B. dentium* ATCC 27678 genome was found to harbor 88 GH-related genes, encoding for 25 different GH families

The *B. dentium* ATCC 27678 genome contains 88 glycosyl hydrolase (GH) genes from 25 different GH families (Fig. 4). The majority of the *B. dentium* ATCC 27678 GH genes are found in families GH3 (14%), GH13 (14%), and GH43 (16%). The GH13 family encodes enzymes which degrade α-glucoside linkages, such as α-amylases [59, 60], while the GH43 family contains xylanase (which break down plant-derived hemicellulose into xylose and arabinose) as well as arabinases (which degrade complex polysaccharides or arabino-oligosaccharides and liberate L-arabinose). The GH3 family notably contains β-glucosidases, β-xylosidases, N-acetylhexosaminidase, and other enzymes. The presence of these GHs suggests a high propensity to degrade dietary plant polysaccharides.

Interestingly, we observed few GHs associated with human milk oligosaccharide (HMOs) or mucin degradation. *B. dentium* did possess genes in GH2 (6.8%; galactosidase); GH 29 (1.1%; fucosidase), and GH125 (1.1%; mannosidase). Surprisingly, *B. dentium* lacked GH33, GH101, GH129, GH84, GH85, GH89, GH95, GH20, and GH38; which are involved in HMO and mucin degradation and common in some *Bifidobacterium* species [42]. Consistent with these findings, the experimental carbohydrate utilization profile (Fig. 3) indicates poor or absent growth on components of host-derived glycans as a sole carbon source, including lactose, N-acetylgalactosamine, N-acetylglucosamine or N-acetylneuraminic acid that would most likely require GH genes from families GH33, GH95, and GH101 (Fig. 3a, d, g, h and i). Consistent with the presence of GH43 and GH125, host-associated galactose and mannose supported growth of *B. dentium* (Fig. 3a), indicating select host factors influence *B. dentium* colonization and growth. As expected, *B. dentium* growth (i.e., a final OD_600nm_ of > 0.2) was also observed on certain plant-derived carbohydrates such as maltose, melibiose, sucrose, ribose, fructose, and turanose (Fig. 3i). These data suggest that in the absence of glucose, *B. dentium* is able to support its growth via 14 different sugars, most of which are plant-derived and may have variable availability depending on the host diet.

### *B. dentium* has limited ability to use amino acids, nucleosides and polymers as a sole carbon source

**Fig. 5:**
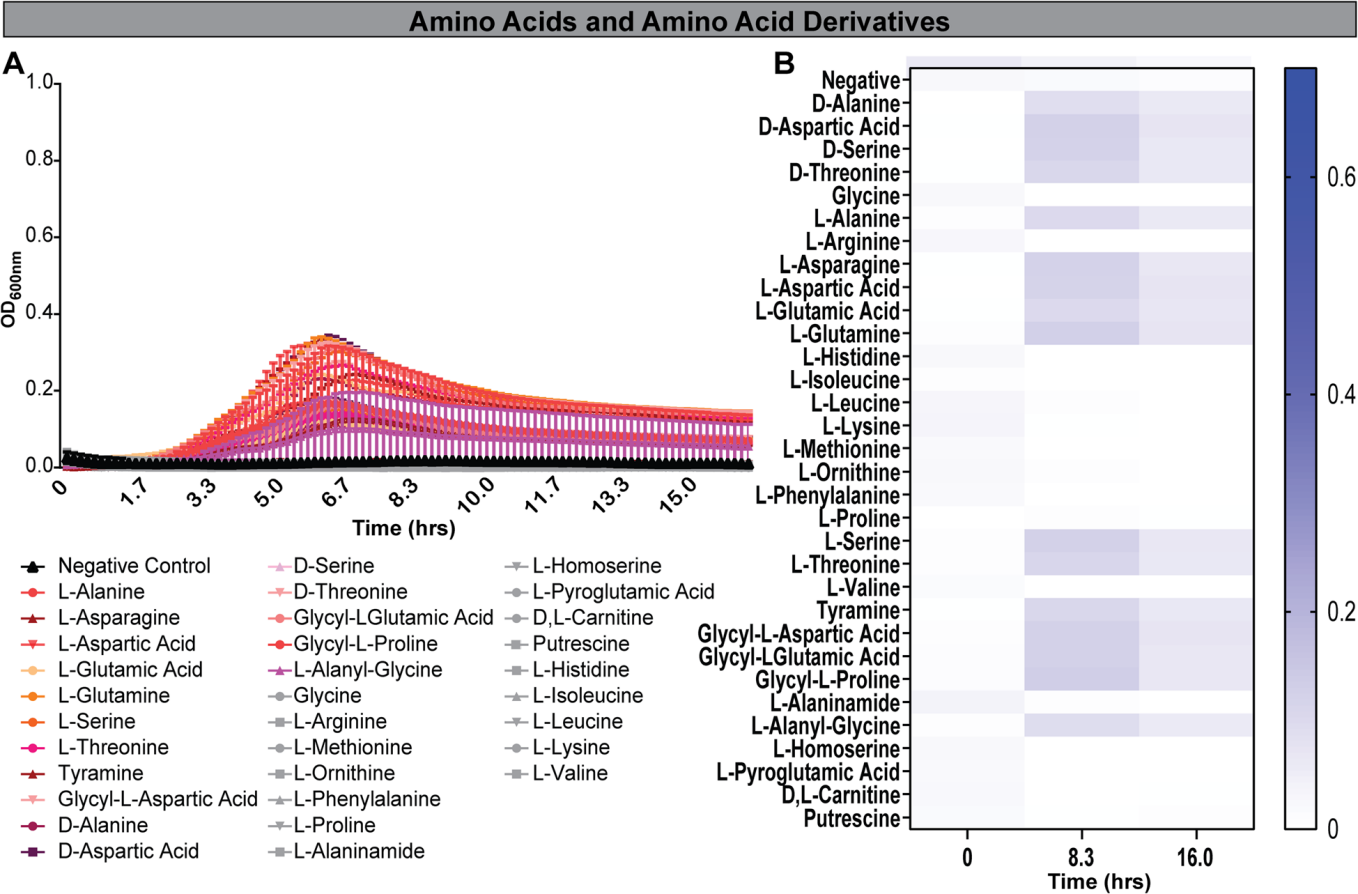
*B. dentium* yields minimal growth on amino acids and amino acid derivatives in LDM4 preparations prepared without glucose. *B. dentium* ATCC 27678 was grown anaerobically at 37 °C in Biolog plates with a fully-defined media (LDM4) preparation that lacked glucose. Growth was monitored over 16 h by plate reader in plate containing (**a**) 33 different amino acids. **b** For visualization, heat maps were generated for all amino acids at time 0, 8.3 and 16.0 h. All data are presented as mean ± stdev

**Fig. 6:**
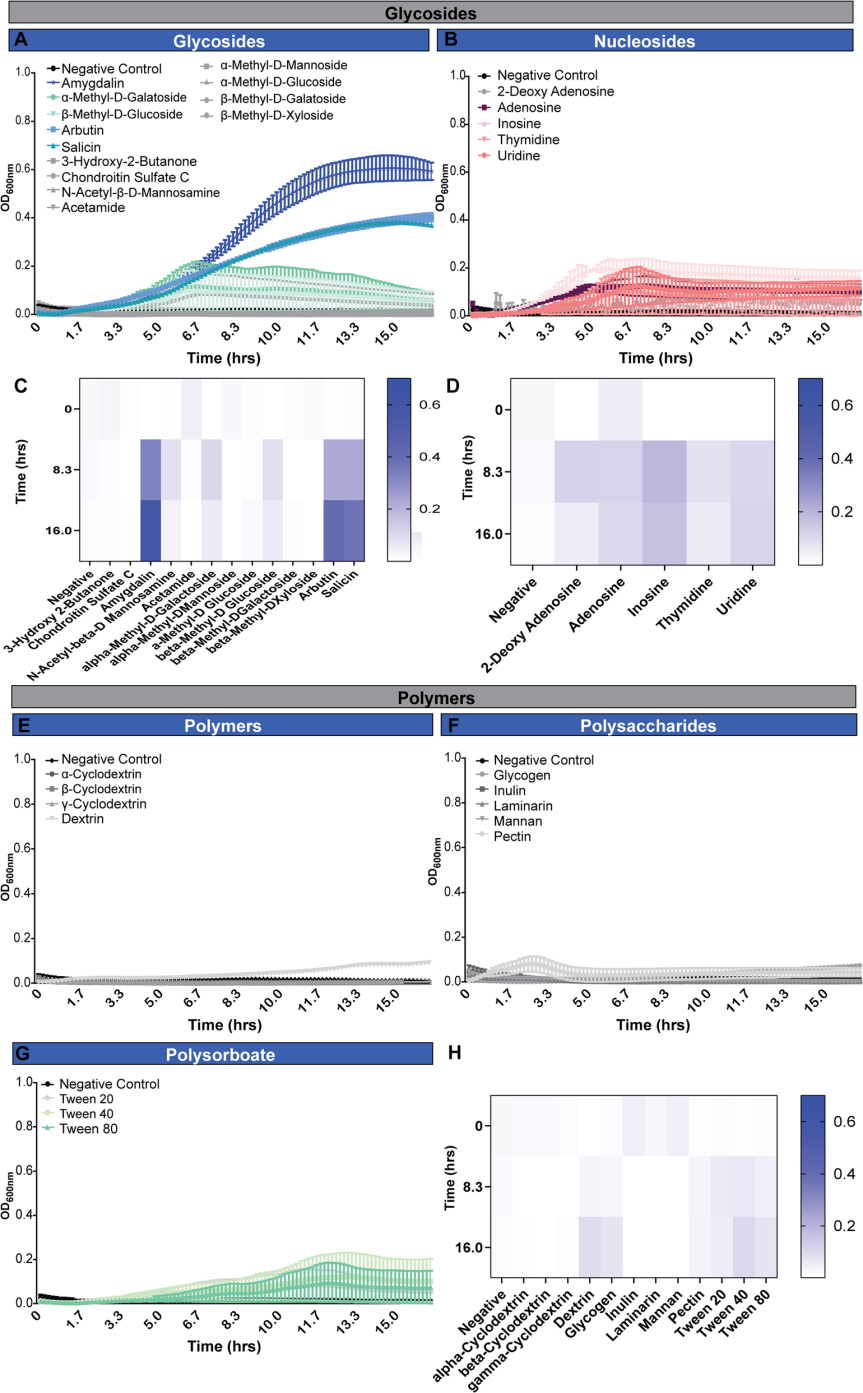
*B. dentium* does not grow on glycosides, nucleosides, polymers, polysaccharides or polysorbates in the absence of glucose, with the exception of amygdalin, arbutin and salicin. *B. dentium* ATCC 27678 was grown anaerobically at 37 °C in Biolog plates with a fully-defined media (LDM4) preparation that lacked glucose. Growth was monitored over 16 h by plate reader in plate containing **a** glycosides, **b** nucleosides. Heat maps were generated for **c** glycosides and **d** nucleosides at time 0, 8.3 and 16 h. *B. dentium* growth was also monitored with **e** polymers, **f** polysaccharides, and **g** polysorbates. **h** For visualization, heat maps were generated for polymers, polysaccharides and polysorbates at time 0, 8.3 and 16.0 h. All data are presented as mean ± stdev

**Fig. 7:**
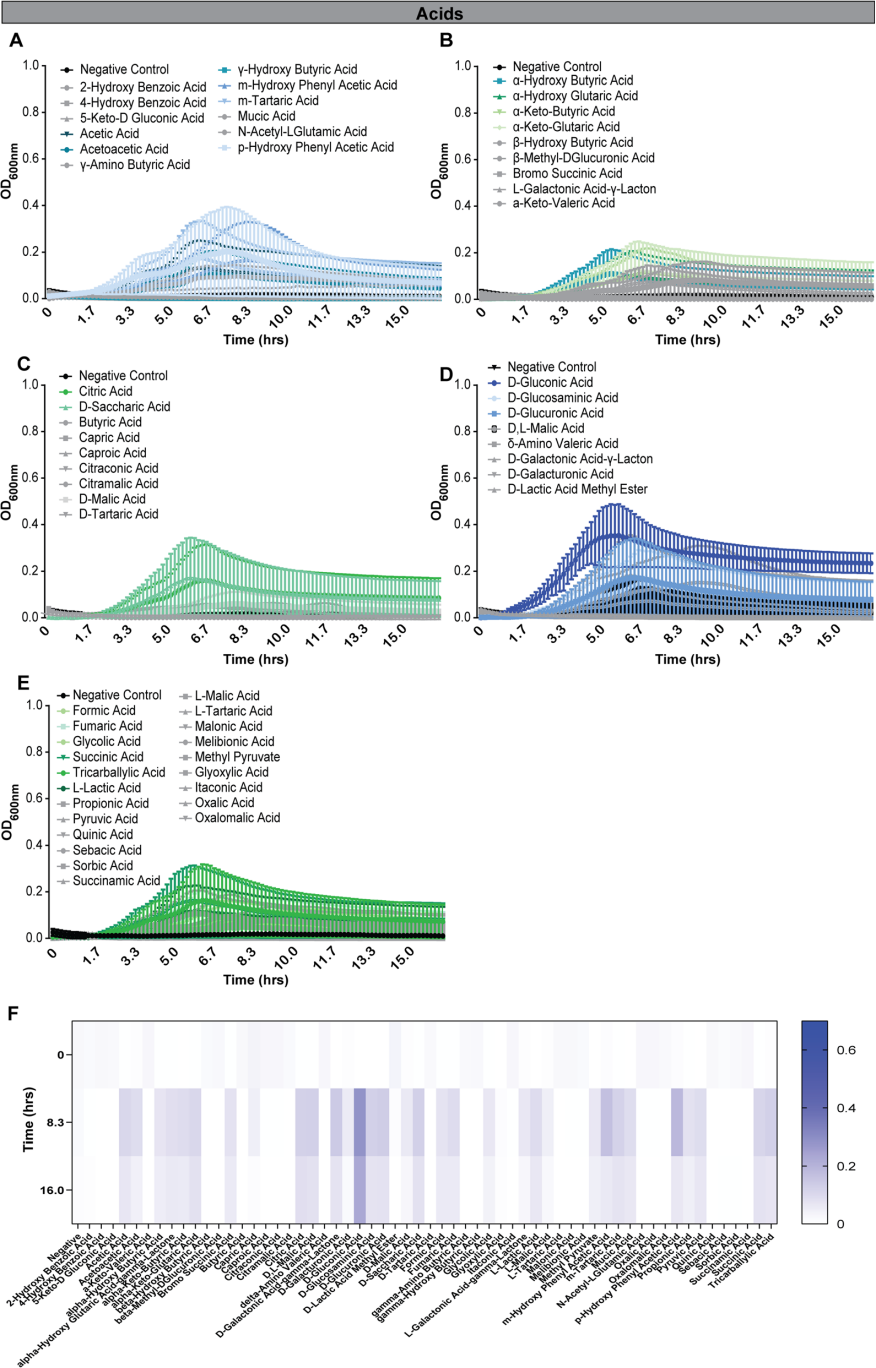
*B. dentium* has minimal growth on organic acids without glucose. *B. dentium* ATCC 27678 was grown anaerobically at 37 °C in Biolog plates with a fully-defined media (LDM4) preparation that lacked glucose. Growth was monitored over 16 h by plate reader in plate containing 59 different organic acids. Acids were separated into groups: **a** 12 acids, **b** 9, **c** 9, **d** 8 and **e** 21 acids. **f** Heat maps were generated for organic acids at time 0, 8.3 and 16.0 h. All data are presented as mean ± stdev

The ability to metabolize peptides and amino acids is a common feature among gut microbiota [61]. However, amino acids and nucleotides are often studied as nitrogen sources rather than a primary carbon source. Currently, little information is available on the ability of bifidobacteria to use these substrates as both primary carbon and nitrogen sources in the absence of additional carbohydrates. We examined the growth of *B. dentium* during a time course on a panel of 32 amino acids and amino acid derivatives in LDM4 lacking glucose (Fig. 5, Tables 3 and 5). Surprisingly, *B. dentium* could use 14 amino acids as sole carbon sources to support limited growth over short time periods ≤8.3 h (OD_600nm_ > 0.2, representing growth) (Fig. 5a, b). These amino acids included D-aspartic acid, D-serine, D-threonine, L-alanine, L-asparagine, L-aspartic acid, L-glutamic acid, L-glutamine, L-serine, L-threonine, tyramine, Glycyl-L-aspartic acid, Glycyl-L-glutamic acid, and Glycyl-L-Proline. Next, we examined *B. dentium* on glycosides and specifically nucleosides (Fig. 6a-d). *B. dentium* had significant growth using amygdalin, arbutin and salicin (Fig. 6a, c), consistent with findings in pigs that these glycosides promote the growth of certain *Bifidobacterium* strains [62]. In contrast, no growth was observed with nucleosides (Fig. 6b, d), cyclodextrin polymers (Fig. 6e, h), or polysorbates (Fig. 6g, h). Interestingly, we observed no significant growth with several polysaccharides (Fig. 6f, h), including inulin, which has been shown in mouse studies and human clinical trials to lead to an increase in bifidobacteria [13, 63–68]. To simulate the diverse number of carbon sources in the GI tract, we supplemented inulin containing LDM4 with glucose and we observed that the combination of carbon sources supported more growth than glucose alone (at 2.5 h: LDM4 glucose control OD_600nm_ = 0.39 ± 0.11, Inulin = 0.54 ± 0.12; mean ± stdev). Finally, we analyzed the ability of *B. dentium* to grow with 59 different organic acid sources. Of the organic acids examined, *B. dentium* growth was only stimulated to statistical significance by D-glucuronic acid (Fig. 7a-f). These data point to the metabolic flexibility of *B. dentium* to use select amino acids, glycosidases and organic acids to support microbial growth in the absence of a carbohydrate source.

**Fig. 8:**
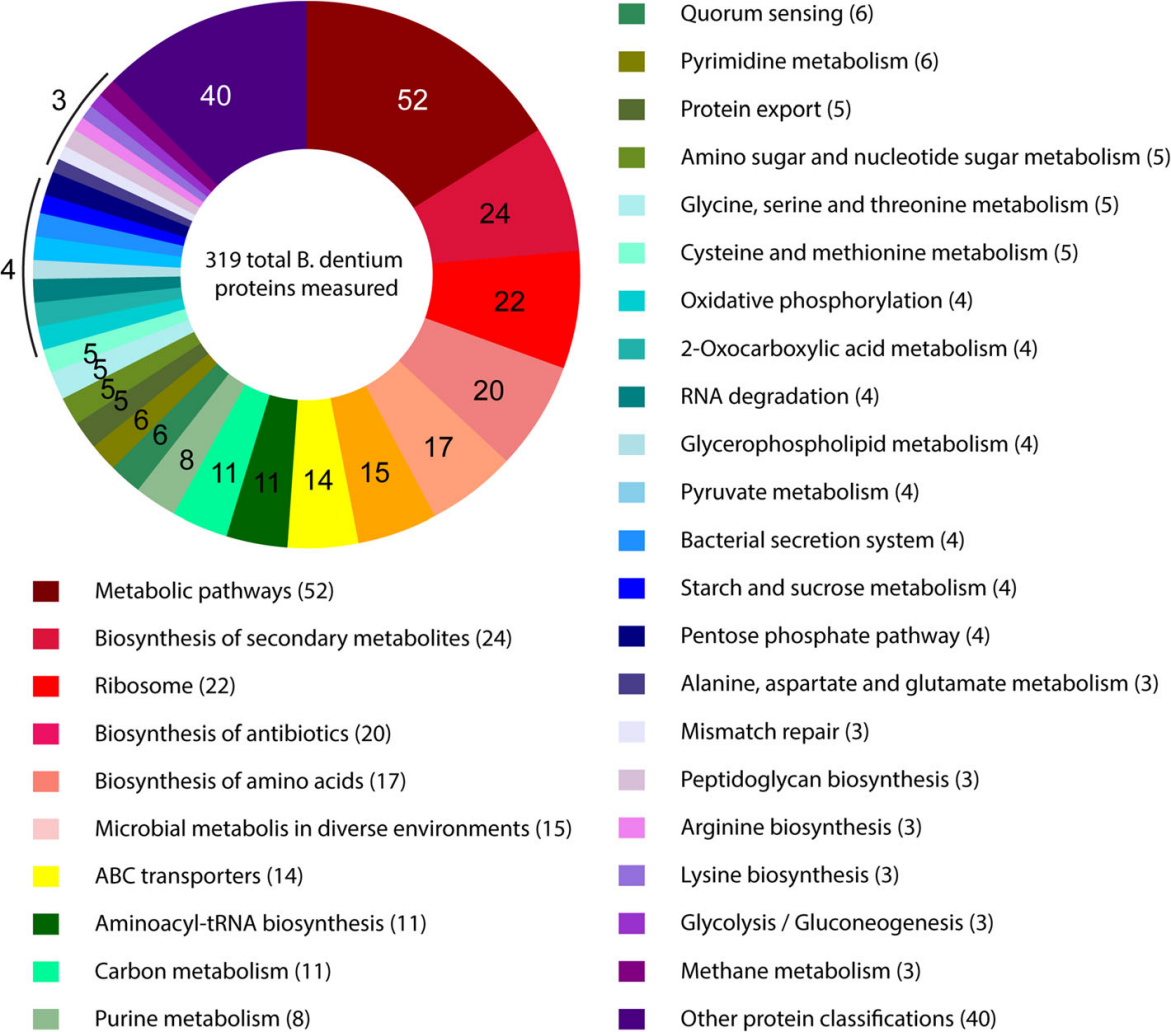
Pathway analysis *B. dentium* by proteomic analysis. *B. dentium* ATCC 27678 were examined using high-resolution liquid chromatography-tandem mass spectrometry based proteomics and 319 proteins were identified from *B. dentium*. The functional classifications of these proteins are illustrated in the pie chart above

These findings of metabolic functionality are supported by our proteomic analysis of *B. dentium* ATCC 27678 from LDM4 cultures using glucose as a primary carbon source (Fig. 8, Table 5). Of the 319 proteins we identified, 52 (16.3%) were involved in metabolic pathways and 15 (4.7%) were involved in metabolism in diverse environments. We observed several proteins involved in carbon metabolism (3.4%), purine metabolism (2.5%), amino sugar and nucleotide sugar metabolism (1.6%), glycine/serine/threonine metabolism (1.6%), cysteine/methionine metabolism (1.6%), pyruvate metabolism (1.3%), starch and sucrose metabolism (1.3%) and alanine/aspartate/glutamate metabolism (0.9%). Consistent with our genome analysis, we observed proteins involved in the pentose phosphate pathway (1.3%) and large number of ABC transporters (4.3%). Together these findings indicate that *B. dentium* can metabolize a wide range of growth substrates, including nutrient sources that are commonly found in the human diet and in the gut lumen.

## Discussion

**Fig. 9:**
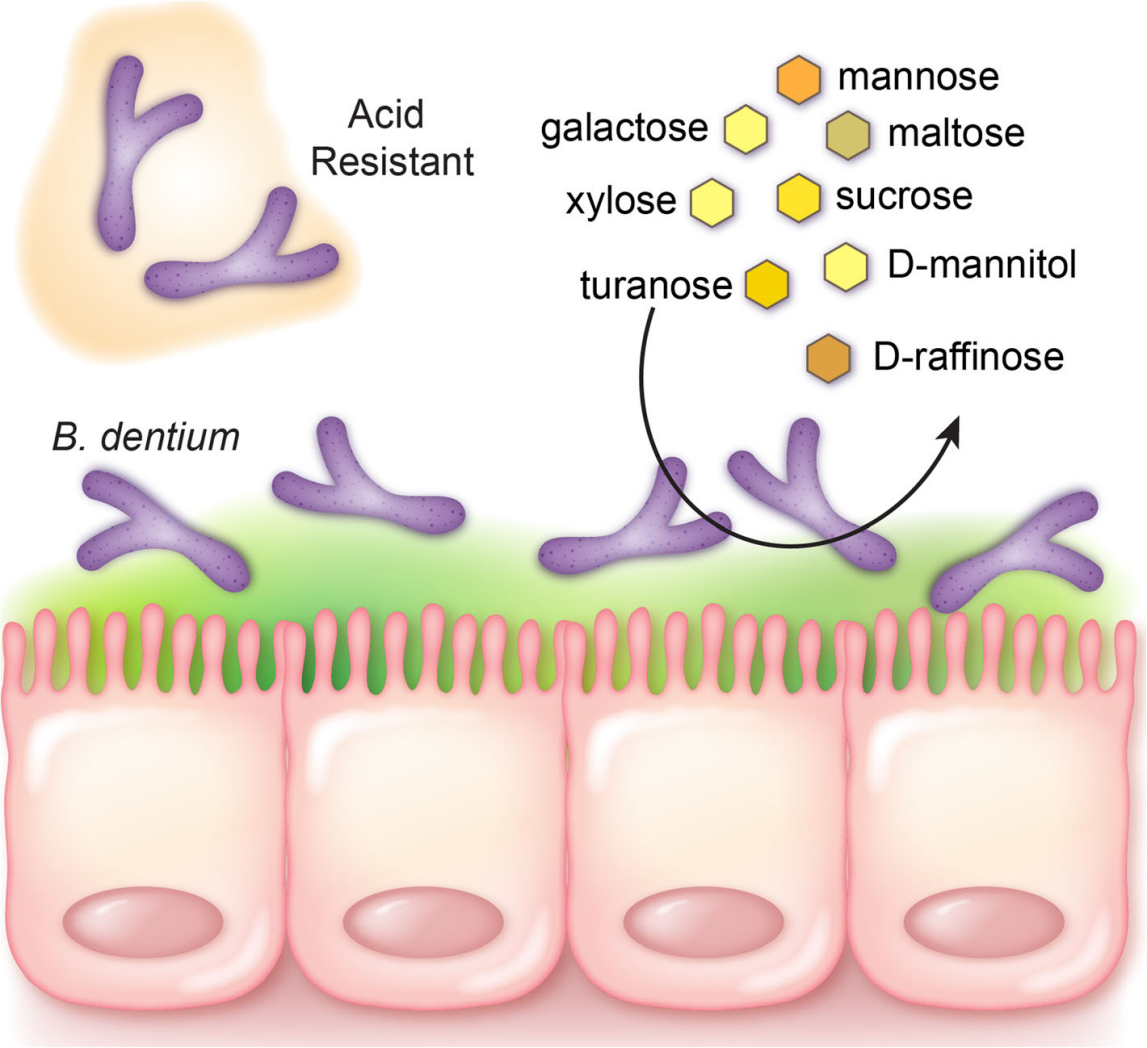
Proposed model for *B. dentium* intestinal colonization. Our data suggest that *B. dentium* is acid resistant, adheres to the intestinal mucus layer and consumes a variety of dietary sources. We speculate that these features contribute to the ability of *B. dentium* to colonize the intestine

The human GI tract is a highly competitive environment characterized by fluctuations in nutrient source availability. As a result, metabolic versatility, which allows microbes to use multiple carbon, nitrogen, and other sources, is characteristic of successful commensal microbes. In this study, we provide an in-depth analysis of *B. dentium* growth in a myriad of conditions, including varying acid conditions and nutrient sources (Fig. 9). We demonstrate that *B. dentium* can survive conditions which mirror the transit through the GI tract and adheres to intestinal mucus, indicating adaptation as a commensal member of the GI tract. The data gathered in this study also provide a substantial amount of information on the growth-promoting properties of *B. dentium*. We demonstrate that in the absence of glucose, *B. dentium* can still use 14 sugars, 4 amino acids/amino acid derivatives/amines, 3 glycosides, and 1 organic acid to support its growth. These data reveal metabolic flexibility in nutrient utilization in *B. dentium*, which likely is key to successful competition in the dynamic intestinal milieu. Despite some carbon sources supporting only modest/or short-term growth, according to Rolf Freter’s nutrient niche hypothesis, we interpret this finding as being both a necessary and a sufficient component of *B. dentium* ecological fitness in the GI tract. It is highly unlikely that long term carbon utilization will depend on any single source in vivo*,* but short-term utilization of variable and transient nutrients is critical to successful colonization [69–71]*.* The data presented demonstrate *B. dentium*’s ability to grow and thrive under varying conditions found in the gastrointestinal tract. These findings enlighten our understanding of the diverse sources that regulate *B. dentium*’s ability to colonize the human intestine.

Like other bifidobacteria, *B. dentium* is a recognized member of the infant and adult intestinal microbiome [3, 6–8, 37]. However, *B. dentium* species are also members of the oral microbiome and have been identified in dental caries [33, 50, 72–81]. In addition to *B. dentium*, *B. breve*, *B. adolescentis*, and *B. longum* have also been isolated from dental caries [33, 50, 72–81]. Although the precise role bifidobacteria plays in dental caries is unknown, *Bifidobacterium* species may be bystanders due to their adhesive properties and their resistance to acidity [50, 82–84]. In gnotobiotic animals, *B. dentium* was found to have beneficial effects on the host, with no adverse effects noted [21–23, 42]; suggesting that *B. dentium* also participates as a commensal intestinal microbe.

Dealing with acid stress is an important factor for colonizing gut microbes. Acid tolerance in bifidobacteria has been linked to the activity of the membrane H + -F1F0-ATPase [53, 85]. The H + -F1F0-ATPase enzyme is responsible for maintaining pH homeostasis in most anaerobic microbes. Acid-resistant *Bifidobacterium* species like *B. animalis* activate the F1F0-ATPase complex upon acid exposure [86, 87]. *B. dentium* encodes the genes for the H + -F1F0-ATPase (KEGG) and based on the relative resistance of *B. dentium* to low pH, we speculate that the H + F1F0-ATPase is likely activated. In *B. longum*, low extracellular pH is reflected by a low intracellular pH [85]. Similar to the literature, our data indicate that *B. dentium*’s intracellular pH can reach a low level without a significant loss in viability. Although these experiments were performed in rich bacterial media, we speculate that *B. dentium* would survive the transit of the gastrointestinal tract. Together, these findings suggest that *B. dentium* harbors compensatory mechanisms to withstand the various pHs of the gastrointestinal tract.

Nutrient availability may be limited in the intestinal lumen due to a variety of factors including competition by other microbes, absorption by the host, or transit through the GI system. Therefore, metabolic plasticity is key to successful microbial colonization. Recent analysis of multiple oral and intestinal derived *B. dentium* genomes identified 140 conserved genes among *B. dentium* strains, indicating a high degree of phylogenetic relatedness [88]. All *B. dentium* genomes shared 19 glycosyl hydrolases families, with the highest abundance observed in GH13. This is consistent with our *B. dentium* ATCC 27678 analysis, which revealed the highest expression of GH13. The glycobiome of *B. dentium* strains also indicated a degradation of a wide range of carbohydrates and plant-derived polysaccharides [88]. Using Biolog phenotyping arrays, we identified that in the absence of glucose, *B. dentium* ATCC 27678 readily uses mannose, xylose, mannitol, maltose, sucrose, melibiose, gentiobiose, trunose, raffinose, maltotriose, and stachyose, Sedoheptulosan. We also observed growth with galactose, which supports previous work indicating that galacto-oligosaccharides (GOS) supplementation bolsters the abundance of bifidobacteria [1, 2, 89]. This is also consistent with the *B. dentium* ATCC 27678 genome, which contains the GH enzyme for β-galactosidases (GH2 and GH42 families), likely allowing *B. dentium* to grow on galacto-oligosaccharides.

Interestingly, we found that in the absence of glucose, *B. dentium* was unable to use several polysaccharides which normally promote bifidobacterial growth. These included well characterized inulin, lactulose, and pectin. Prebiotic substrates, in particular inulin and lactulose, have been used in human trials where they have been observed to increase *Bifidobacterium* spp. and provide beneficial effects to the host [13, 36, 90–93]. The addition of glucose back into the LDM4 preparation in our studies showed that *B. dentium* ATCC 27678 growth was enhanced with inulin, confirming the dependence on glucose for inulin metabolism [63].

We also observed that in the absence of glucose*, B. dentium* was able to use amino acids to support baseline growth. Limited data are available on nitrogen assimilation in the gut lumen, particularly by *Bifidobacterium* species [94]. Herein, we provide evidence for metabolism of select amino acids in lieu of a carbohydrate-based carbon source which can be used as carbon or nitrogen substrates as needed. We also found that *B. dentium* was largely unable to use host glycan sugars. This finding is consistent with the GH profile of *B. dentium* and our previous work which found that *B. dentium* could not degrade intact MUC2 mucus [42]. Other bifidobacteria, such as *B. bifidum* (PRL2010, D119 and L22), *B. breve* NCIMB8807, and *B. longum* NCIMB8809, harbor a much larger repertoire of mucin-degrading glycosyl hydrolases [95–99]. These mucin- and HMO-degrading GHs likely provide these *Bifidobacterium* strains with a competitive edge, allowing these microbes to be found at greater abundance than *B. dentium* in vivo.

Consistent with GHs profile, we found that *B. dentium* exhibited substantial growth on β-glucans. *B. dentium* harbors GH 1, 3 and 30 which harbor β-glucosidases which can degrade plant based β-glucans and natural phenols, such as salicin, arbutin and amygdalin. Additionally, *B. dentium* had robust growth on these β-glucans, indicating that *B. dentium* may target plant-based nutrients. Our in vitro findings indicate that *B. dentium* supports it growth with several plant-derived compounds which closely mirrors dietary studies in humans. For example, consumption of pea and whey protein extract increases bifidobacteria levels in healthy subjects [100–102]. Consumption of date fruits, containing high levels of glucose, fructose, and sucrose, has also been reported to increase the relative abundance of bifidobacteria [103–105]. Diets rich in non-digestible carbohydrates, such as whole grain and wheat bran, are also linked to increases in bifidobacteria [106, 107]. In contrast, a Western diet (high in animal protein and fat, low in fiber) has been associated with decreased bifidobacteria [108–110]. These human studies support the important role of dietary compounds in modulating the microbial community and influencing the levels of bifidobacteria. Bifidobacteria have been associated with numerous health benefits, including immune-modulation, gut-brain-axis cross-talk, increasing intestinal mucus, enhancing epithelial integrity, pathogen exclusion, cancer prevention, and management of inflammatory bowel disease [16, 21, 23, 38, 42, 93, 111–143]. Thus, maintenance of bifidobacteria is likely important for maintaining intestinal homeostasis. Based on our newly identified nutrient sources for *B. dentium*, we propose that these compounds could be implemented in the future to promote *B. dentium* abundance in the human gastrointestinal tract. Collectively this work provides novel insights into the proteome and metabolic profile of *B. dentium* and our findings point to *B. dentium* as a well-adapted member of the gastrointestinal tract.

## Supplementary Information

**Additional file 1.**

## Data Availability

The datasets generated in the current study are deposited in the NIH-funded Center for Computational Mass Spectrometry MassIVE database, Dataset Number MSV000086294. Link: massive.ucsd.edu/ProteoSAFe/private-dataset.jsp?task=9e9343b12d3540e7bc8c470f2de719d3.

## References

[1] Ben XM, Li J, Feng ZT, Shi SY, Lu YD, Chen R, Zhou XY (2008). Low level of galacto-oligosaccharide in infant formula stimulates growth of intestinal Bifidobacteria and lactobacilli. World J Gastroenterol.

[2] Fanaro S, Marten B, Bagna R, Vigi V, Fabris C, Pena-Quintana L (2009). Galacto-oligosaccharides are bifidogenic and safe at weaning: a double-blind randomized multicenter study. J Pediatr Gastroenterol Nutr.

[3] He M, Li M, Wang SY, Zhang LL, Miao JJ, Shi L, Yu Q, Yao JR, Huang CY, He F (2016). Analyzing colonization of Bifidobacteria in infants with real-time fluorescent quantitative PCR. Sichuan Da Xue Xue Bao Yi Xue Ban.

[4] Kirmiz N, Robinson RC, Shah IM, Barile D, Mills DA (2018). Milk Glycans and their interaction with the infant-gut microbiota. Annu Rev Food Sci Technol.

[5] Lee JH, O'Sullivan DJ (2010). Genomic insights into bifidobacteria. Microbiol Mol Biol Rev.

[6] Nagpal R, Kurakawa T, Tsuji H, Takahashi T, Kawashima K, Nagata S, Nomoto K, Yamashiro Y (2017). Evolution of gut Bifidobacterium population in healthy Japanese infants over the first three years of life: a quantitative assessment. Sci Rep.

[7] Rinne MM, Gueimonde M, Kalliomaki M, Hoppu U, Salminen SJ, Isolauri E (2005). Similar bifidogenic effects of prebiotic-supplemented partially hydrolyzed infant formula and breastfeeding on infant gut microbiota. FEMS Immunol Med Microbiol.

[8] Turroni F, Peano C, Pass DA, Foroni E, Severgnini M, Claesson MJ, Kerr C, Hourihane J, Murray D, Fuligni F, Gueimonde M, Margolles A, de Bellis G, O’Toole PW, van Sinderen D, Marchesi JR, Ventura M (2012). Diversity of bifidobacteria within the infant gut microbiota. PLoS One.

[9] Turroni F, Marchesi JR, Foroni E, Gueimonde M, Shanahan F, Margolles A, van Sinderen D, Ventura M (2009). Microbiomic analysis of the bifidobacterial population in the human distal gut. ISME J.

[10] Qin J, Li R, Raes J, Arumugam M, Burgdorf KS, Manichanh C (2010). A human gut microbial gene catalogue established by metagenomic sequencing. Nature..

[11] Flint HJ, Bayer EA, Rincon MT, Lamed R, White BA (2008). Polysaccharide utilization by gut bacteria: potential for new insights from genomic analysis. Nat Rev Microbiol..

[12] Jasberg H, Soderling E, Endo A, Beighton D, Haukioja A (2016). Bifidobacteria inhibit the growth of Porphyromonas gingivalis but not of Streptococcus mutans in an in vitro biofilm model. Eur J Oral Sci.

[13] Kolida S, Tuohy K, Gibson GR (2002). Prebiotic effects of inulin and oligofructose. Br J Nutr.

[14] O'Callaghan A, van Sinderen D (2016). Bifidobacteria and their role as members of the human gut microbiota. Front Microbiol.

[15] Vazquez-Gutierrez P, de Wouters T, Werder J, Chassard C, Lacroix C (2016). High iron-sequestrating Bifidobacteria inhibit Enteropathogen growth and adhesion to intestinal epithelial cells in vitro. Front Microbiol.

[16] Fukuda S, Toh H, Taylor TD, Ohno H, Hattori M (2012). Acetate-producing bifidobacteria protect the host from enteropathogenic infection via carbohydrate transporters. Gut Microbes.

[17] Bohm S, Kruis W (2006). Probiotics in chronic inflammatory bowel disease. MMW Fortschr Med.

[18] Broekaert IJ, Walker WA (2006). Probiotics and chronic disease. J Clin Gastroenterol.

[19] Yatsunenko T, Rey FE, Manary MJ, Trehan I, Dominguez-Bello MG, Contreras M, Magris M, Hidalgo G, Baldassano RN, Anokhin AP, Heath AC, Warner B, Reeder J, Kuczynski J, Caporaso JG, Lozupone CA, Lauber C, Clemente JC, Knights D, Knight R, Gordon JI (2012). Human gut microbiome viewed across age and geography. Nature..

[20] Cheikhyoussef A, Pogori N, Chen W, Zhang H (2008). Antimicrobial proteinaceous compounds obtained from bifidobacteria: from production to their application. Int J Food Microbiol.

[21] Luk B, Veeraragavan S, Engevik M, Balderas M, Major A, Runge J, Luna RA, Versalovic J (2018). Postnatal colonization with human “infant-type” Bifidobacterium species alters behavior of adult gnotobiotic mice. PLoS One.

[22] Engevik MA, Luck B, Visuthranukul C, Ihekweazu FD, Engevik AC, Shi Z, Danhof HA, Chang-Graham AL, Hall A, Endres BT, Haidacher SJ, Horvath TD, Haag AM, Devaraj S, Garey KW, Britton RA, Hyser JM, Shroyer NF, Versalovic J (2020). Human-derived Bifidobacterium dentium modulates the mammalian serotonergic system and gut-brain axis. Cell Mol Gastroenterol Hepatol.

[23] Luck B, Engevik MA, Ganesh BP, Lackey EP, Lin T, Balderas M, Major A, Runge J, Luna RA, Sillitoe RV, Versalovic J (2020). Bifidobacteria shape host neural circuits during postnatal development by promoting synapse formation and microglial function. Sci Rep.

[24] Martens EC, Chiang HC, Gordon JI (2008). Mucosal glycan foraging enhances fitness and transmission of a saccharolytic human gut bacterial symbiont. Cell Host Microbe.

[25] Ventura M, O'Flaherty S, Claesson MJ, Turroni F, Klaenhammer TR, van Sinderen D, O'Toole PW (2009). Genome-scale analyses of health-promoting bacteria: probiogenomics. Nat Rev Microbiol.

[26] Zoetendal EG, Raes J, van den Bogert B, Arumugam M, Booijink CC, Troost FJ (2012). The human small intestinal microbiota is driven by rapid uptake and conversion of simple carbohydrates. ISME J..

[27] Nicholson JK, Holmes E, Kinross J, Burcelin R, Gibson G, Jia W, Pettersson S (2012). Host-gut microbiota metabolic interactions. Science..

[28] Lacroix C, de Wouters T, Chassard C (2015). Integrated multi-scale strategies to investigate nutritional compounds and their effect on the gut microbiota. Curr Opin Biotechnol.

[29] Salonen A, de Vos WM (2014). Impact of diet on human intestinal microbiota and health. Annu Rev Food Sci Technol.

[30] Turroni F, Bottacini F, Foroni E, Mulder I, Kim JH, Zomer A, Sanchez B, Bidossi A, Ferrarini A, Giubellini V, Delledonne M, Henrissat B, Coutinho P, Oggioni M, Fitzgerald GF, Mills D, Margolles A, Kelly D, van Sinderen D, Ventura M (2010). Genome analysis of Bifidobacterium bifidum PRL2010 reveals metabolic pathways for host-derived glycan foraging. Proc Natl Acad Sci U S A.

[31] Lee JH, Karamychev VN, Kozyavkin SA, Mills D, Pavlov AR, Pavlova NV, Polouchine NN, Richardson PM, Shakhova VV, Slesarev AI, Weimer B, O'Sullivan DJ (2008). Comparative genomic analysis of the gut bacterium Bifidobacterium longum reveals loci susceptible to deletion during pure culture growth. BMC Genomics.

[32] O'Connell Motherway M, Zomer A, Leahy SC, Reunanen J, Bottacini F, Claesson MJ, O'Brien F, Flynn K, Casey PG, Moreno Munoz JA, Kearney B, Houston AM, O'Mahony C, Higgins DG, Shanahan F, Palva A, de Vos WM, Fitzgerald GF, Ventura M, O'Toole PW, van Sinderen D (2011). Functional genome analysis of Bifidobacterium breve UCC2003 reveals type IVb tight adherence (Tad) pili as an essential and conserved host-colonization factor. Proc Natl Acad Sci U S A.

[33] Ventura M, Turroni F, Zomer A, Foroni E, Giubellini V, Bottacini F, Canchaya C, Claesson MJ, He F, Mantzourani M, Mulas L, Ferrarini A, Gao B, Delledonne M, Henrissat B, Coutinho P, Oggioni M, Gupta RS, Zhang Z, Beighton D, Fitzgerald GF, O'Toole PW, van Sinderen D (2009). The Bifidobacterium dentium Bd1 genome sequence reflects its genetic adaptation to the human oral cavity. PLoS Genet.

[34] Schell MA, Karmirantzou M, Snel B, Vilanova D, Berger B, Pessi G, Zwahlen MC, Desiere F, Bork P, Delley M, Pridmore RD, Arigoni F (2002). The genome sequence of Bifidobacterium longum reflects its adaptation to the human gastrointestinal tract. Proc Natl Acad Sci U S A.

[35] Barrangou R, Briczinski EP, Traeger LL, Loquasto JR, Richards M, Horvath P, Coûté-Monvoisin AC, Leyer G, Rendulic S, Steele JL, Broadbent JR, Oberg T, Dudley EG, Schuster S, Romero DA, Roberts RF (2009). Comparison of the complete genome sequences of Bifidobacterium animalis subsp. lactis DSM 10140 and Bl-04. J Bacteriol.

[36] Watson D, O'Connell Motherway M, Schoterman MH, van Neerven RJ, Nauta A, van Sinderen D (2013). Selective carbohydrate utilization by lactobacilli and bifidobacteria. J Appl Microbiol.

[37] Human Microbiome Project C (2012). Structure, function and diversity of the healthy human microbiome. Nature..

[38] Pokusaeva K, Johnson C, Luk B, Uribe G, Fu Y, Oezguen N, et al. GABA-producing Bifidobacterium dentium modulates visceral sensitivity in the intestine. Neurogastroenterol Motil. 2017;29(1). 10.1111/nmo.12904. 10.1111/nmo.12904 PMC5195897 27458085

[39] Nebra Y, Bonjoch X, Blanch AR (2003). Use of Bifidobacterium dentium as an indicator of the origin of fecal water pollution. Appl Environ Microbiol.

[40] Menard O, Butel MJ, Gaboriau-Routhiau V, Waligora-Dupriet AJ (2008). Gnotobiotic mouse immune response induced by Bifidobacterium sp. strains isolated from infants. Appl Environ Microbiol.

[41] Ventura M, Elli M, Reniero R, Zink R (2001). Molecular microbial analysis of Bifidobacterium isolates from different environments by the species-specific amplified ribosomal DNA restriction analysis (ARDRA). FEMS Microbiol Ecol.

[42] Engevik MA, Luk B, Chang-Graham AL, Hall A, Herrmann B, Ruan W, et al. Bifidobacterium dentium fortifies the intestinal mucus layer via autophagy and calcium signaling pathways. mBio. 2019;10(3). 10.1128/mBio.01087-19. 10.1128/mBio.01087-19 PMC6581858 31213556

[43] Hall AE, Engevik MA, Oezguen N, Haag A, Versalovic J (2019). ClC transporter activity modulates histidine catabolism in Lactobacillus reuteri by altering intracellular pH and membrane potential. Microb Cell Factories.

[44] Engevik MA, Morra CN, Roth D, Engevik K, Spinler JK, Devaraj S (2019). Microbial metabolic capacity for intestinal folate production and modulation of host folate receptors. Front Microbiol.

[45] Marchler-Bauer A, Bo Y, Han L, He J, Lanczycki CJ, Lu S, Chitsaz F, Derbyshire MK, Geer RC, Gonzales NR, Gwadz M, Hurwitz DI, Lu F, Marchler GH, Song JS, Thanki N, Wang Z, Yamashita RA, Zhang D, Zheng C, Geer LY, Bryant SH (2017). CDD/SPARCLE: functional classification of proteins via subfamily domain architectures. Nucleic Acids Res.

[46] Marchler-Bauer A, Derbyshire MK, Gonzales NR, Lu S, Chitsaz F, Geer LY, Geer RC, He J, Gwadz M, Hurwitz DI, Lanczycki CJ, Lu F, Marchler GH, Song JS, Thanki N, Wang Z, Yamashita RA, Zhang D, Zheng C, Bryant SH (2015). CDD: NCBI's conserved domain database. Nucleic Acids Res.

[47] Marchler-Bauer A, Lu S, Anderson JB, Chitsaz F, Derbyshire MK, DeWeese-Scott C, Fong JH, Geer LY, Geer RC, Gonzales NR, Gwadz M, Hurwitz DI, Jackson JD, Ke Z, Lanczycki CJ, Lu F, Marchler GH, Mullokandov M, Omelchenko MV, Robertson CL, Song JS, Thanki N, Yamashita RA, Zhang D, Zhang N, Zheng C, Bryant SH (2011). CDD: a conserved domain database for the functional annotation of proteins. Nucleic Acids Res.

[48] Marchler-Bauer A, Bryant SH (2004). CD-Search: protein domain annotations on the fly. Nucleic Acids Res.

[49] Maus JE, Ingham SC (2003). Employment of stressful conditions during culture production to enhance subsequent cold- and acid-tolerance of bifidobacteria. J Appl Microbiol.

[50] Nakajo K, Takahashi N, Beighton D (2010). Resistance to acidic environments of caries-associated bacteria: Bifidobacterium dentium and Bifidobacterium longum. Caries Res.

[51] Jiang Y, Ren F, Liu S, Zhao L, Guo H, Hou C (2016). Enhanced acid tolerance in Bifidobacterium longum by adaptive evolution: comparison of the genes between the acid-resistant variant and wild-type strain. J Microbiol Biotechnol.

[52] Sanchez B, Champomier-Verges MC, Collado Mdel C, Anglade P, Baraige F, Sanz Y (2007). Low-pH adaptation and the acid tolerance response of Bifidobacterium longum biotype longum. Appl Environ Microbiol.

[53] Matsumoto M, Ohishi H, Benno Y (2004). H+-ATPase activity in Bifidobacterium with special reference to acid tolerance. Int J Food Microbiol.

[54] Kainulainen V, Reunanen J, Hiippala K, Guglielmetti S, Vesterlund S, Palva A, Satokari R (2013). BopA does not have a major role in the adhesion of Bifidobacterium bifidum to intestinal epithelial cells, extracellular matrix proteins, and mucus. Appl Environ Microbiol.

[55] Lebeer S, Vanderleyden J, De Keersmaecker SC (2010). Host interactions of probiotic bacterial surface molecules: comparison with commensals and pathogens. Nat Rev Microbiol..

[56] Ewaschuk JB, Diaz H, Meddings L, Diederichs B, Dmytrash A, Backer J, Looijer-van Langen M, Madsen KL (2008). Secreted bioactive factors from Bifidobacterium infantis enhance epithelial cell barrier function. Am J Physiol Gastrointest Liver Physiol.

[57] Maynard CL, Elson CO, Hatton RD, Weaver CT (2012). Reciprocal interactions of the intestinal microbiota and immune system. Nature..

[58] Schwab C, Ruscheweyh HJ, Bunesova V, Pham VT, Beerenwinkel N, Lacroix C (2017). Trophic interactions of infant Bifidobacteria and Eubacterium hallii during L-fucose and fucosyllactose degradation. Front Microbiol.

[59] Svensson B (1994). Protein engineering in the alpha-amylase family: catalytic mechanism, substrate specificity, and stability. Plant Mol Biol.

[60] Janecek S (1997). Alpha-amylase family: molecular biology and evolution. Prog Biophys Mol Biol.

[61] Davila AM, Blachier F, Gotteland M, Andriamihaja M, Benetti PH, Sanz Y, Tomé D (2013). Intestinal luminal nitrogen metabolism: role of the gut microbiota and consequences for the host. Pharmacol Res.

[62] Mikkelsen LL, Bendixen C, Jakobsen M, Jensen BB (2003). Enumeration of bifidobacteria in gastrointestinal samples from piglets. Appl Environ Microbiol.

[63] Rossi M, Corradini C, Amaretti A, Nicolini M, Pompei A, Zanoni S, Matteuzzi D (2005). Fermentation of fructooligosaccharides and inulin by bifidobacteria: a comparative study of pure and fecal cultures. Appl Environ Microbiol.

[64] Bouhnik Y, Flourie B, Riottot M, Bisetti N, Gailing MF, Guibert A (1996). Effects of fructo-oligosaccharides ingestion on fecal bifidobacteria and selected metabolic indexes of colon carcinogenesis in healthy humans. Nutr Cancer.

[65] Bouhnik Y, Vahedi K, Achour L, Attar A, Salfati J, Pochart P (1999). Short-chain fructo-oligosaccharide administration dose-dependently increases fecal bifidobacteria in healthy humans. J Nutr.

[66] Gibson GR, Beatty ER, Wang X, Cummings JH (1995). Selective stimulation of bifidobacteria in the human colon by oligofructose and inulin. Gastroenterology..

[67] Roller M, Clune Y, Collins K, Rechkemmer G, Watzl B (2007). Consumption of prebiotic inulin enriched with oligofructose in combination with the probiotics Lactobacillus rhamnosus and Bifidobacterium lactis has minor effects on selected immune parameters in polypectomised and colon cancer patients. Br J Nutr.

[68] Ramirez-Farias C, Slezak K, Fuller Z, Duncan A, Holtrop G, Louis P (2009). Effect of inulin on the human gut microbiota: stimulation of Bifidobacterium adolescentis and Faecalibacterium prausnitzii. Br J Nutr.

[69] Fabich AJ, Jones SA, Chowdhury FZ, Cernosek A, Anderson A, Smalley D, McHargue JW, Hightower GA, Smith JT, Autieri SM, Leatham MP, Lins JJ, Allen RL, Laux DC, Cohen PS, Conway T (2008). Comparison of carbon nutrition for pathogenic and commensal Escherichia coli strains in the mouse intestine. Infect Immun.

[70] Freter R, Fuller R (1992). Factors affecting the microecology of the gut. Probiotics: the scientific basis.

[71] Stecher B, Hardt WD (2011). Mechanisms controlling pathogen colonization of the gut. Curr Opin Microbiol.

[72] Becker MR, Paster BJ, Leys EJ, Moeschberger ML, Kenyon SG, Galvin JL, Boches SK, Dewhirst FE, Griffen AL (2002). Molecular analysis of bacterial species associated with childhood caries. J Clin Microbiol.

[73] Eriksson L, Lif Holgerson P, Johansson I (2017). Saliva and tooth biofilm bacterial microbiota in adolescents in a low caries community. Sci Rep.

[74] Crociani F, Biavati B, Alessandrini A, Chiarini C, Scardovi V (1996). Bifidobacterium inopinatum sp. nov. and Bifidobacterium denticolens sp. nov., two new species isolated from human dental caries. Int J Syst Bacteriol.

[75] Modesto M, Biavati B, Mattarelli P (2006). Occurrence of the family bifidobacteriaceae in human dental caries and plaque. Caries Res.

[76] Hojo K, Nagaoka S, Murata S, Taketomo N, Ohshima T, Maeda N (2007). Reduction of vitamin K concentration by salivary Bifidobacterium strains and their possible nutritional competition with Porphyromonas gingivalis. J Appl Microbiol.

[77] Edwardsson S (1974). Bacteriological studies on deep areas of carious dentine. Odontol Revy Suppl.

[78] Sanyal B, Russell C (1978). Nonsporing, anaerobic, gram-positive rods in saliva and the gingival crevice of humans. Appl Environ Microbiol.

[79] Maeda N (1980). Anaerobic, gram-positive, pleomorphic rods in human gingival crevice. Bull Tokyo Med Dent Univ.

[80] Moore WE, Holdeman LV, Cato EP, Good IJ, Smith EP, Ranney RR, Palcanis KG (1984). Variation in periodontal floras. Infect Immun.

[81] Mantzourani M, Gilbert SC, Sulong HN, Sheehy EC, Tank S, Fenlon M (2009). The isolation of bifidobacteria from occlusal carious lesions in children and adults. Caries Res.

[82] Aas JA, Griffen AL, Dardis SR, Lee AM, Olsen I, Dewhirst FE, Leys EJ, Paster BJ (2008). Bacteria of dental caries in primary and permanent teeth in children and young adults. J Clin Microbiol.

[83] Utto P, Teanpaisan R, Piwat S, Chandeying V (2017). Assessment of prevalence, adhesion and surface charges of Bifidobacterium spp. isolated from Thai women with bacterial Vaginosis and healthy women. J Med Assoc Thail.

[84] Utto P, Piwat S, Teanpaisan R (2016). Prevalence and adhesion properties of oral Bifidobacterium species in caries-active and caries-free Thai children. Walailak J.

[85] Takahashi N, Xiao JZ, Miyaji K, Yaeshiima T, Hiramatsu A, Iwatsuki K, Kokubo S, Hosono A (2004). Selection of acid tolerant bifidobacteria and evidence for a low-pH-inducible acid tolerance response in Bifidobacterium longum. J Dairy Res.

[86] Ventura M, Canchaya C, van Sinderen D, Fitzgerald GF, Zink R (2004). Bifidobacterium lactis DSM 10140: identification of the atp (atpBEFHAGDC) operon and analysis of its genetic structure, characteristics, and phylogeny. Appl Environ Microbiol.

[87] Sanchez B, de los Reyes-Gavilan CG, Margolles A (2006). The F1F0-ATPase of Bifidobacterium animalis is involved in bile tolerance. Environ Microbiol.

[88] Lugli GA, Tarracchini C, Alessandri G, Milani C, Mancabelli L, Turroni F, et al. Decoding the genomic variability among members of the Bifidobacterium dentium species. Microorganisms. 2020;8(11). 10.3390/microorganisms8111720. 10.3390/microorganisms8111720 PMC7693768 33152994

[89] Maathuis AJ, van den Heuvel EG, Schoterman MH, Venema K (2012). Galacto-oligosaccharides have prebiotic activity in a dynamic in vitro colon model using a (13) C-labeling technique. J Nutr.

[90] Menne E, Guggenbuhl N, Roberfroid M (2000). Fn-type chicory inulin hydrolysate has a prebiotic effect in humans. J Nutr.

[91] Bosscher D, Van Loo J, Franck A (2006). Inulin and oligofructose as prebiotics in the prevention of intestinal infections and diseases. Nutr Res Rev.

[92] Davis LM, Martinez I, Walter J, Hutkins R (2010). A dose dependent impact of prebiotic galactooligosaccharides on the intestinal microbiota of healthy adults. Int J Food Microbiol.

[93] Veereman-Wauters G, Staelens S, Van de Broek H, Plaskie K, Wesling F, Roger LC (2011). Physiological and bifidogenic effects of prebiotic supplements in infant formulae. J Pediatr Gastroenterol Nutr.

[94] Ferrario C, Duranti S, Milani C, Mancabelli L, Lugli GA, Turroni F, Mangifesta M, Viappiani A, Ossiprandi MC, van Sinderen D, Ventura M (2015). Exploring amino acid Auxotrophy in Bifidobacterium bifidum PRL2010. Front Microbiol.

[95] Salyers AA, West SE, Vercellotti JR, Wilkins TD (1977). Fermentation of mucins and plant polysaccharides by anaerobic bacteria from the human colon. Appl Environ Microbiol.

[96] Ruas-Madiedo P, Gueimonde M, Fernandez-Garcia M, de los Reyes-Gavilan CG, Margolles A (2008). Mucin degradation by Bifidobacterium strains isolated from the human intestinal microbiota. Appl Environ Microbiol.

[97] Bayliss CE, Houston AP (1984). Characterization of plant polysaccharide- and mucin-fermenting anaerobic bacteria from human feces. Appl Environ Microbiol.

[98] Abe F, Muto M, Yaeshima T, Iwatsuki K, Aihara H, Ohashi Y, Fujisawa T (2010). Safety evaluation of probiotic bifidobacteria by analysis of mucin degradation activity and translocation ability. Anaerobe..

[99] Egan M, Motherway MO, Kilcoyne M, Kane M, Joshi L, Ventura M (2014). Cross-feeding by Bifidobacterium breve UCC2003 during co-cultivation with Bifidobacterium bifidum PRL2010 in a mucin-based medium. BMC Microbiol.

[100] Swiatecka D, Narbad A, Ridgway KP, Kostyra H (2011). The study on the impact of glycated pea proteins on human intestinal bacteria. Int J Food Microbiol.

[101] Meddah AT, Yazourh A, Desmet I, Risbourg B, Verstraete W, Romond MB (2001). The regulatory effects of whey retentate from bifidobacteria fermented milk on the microbiota of the simulator of the human intestinal microbial ecosystem (SHIME). J Appl Microbiol.

[102] Romond MB, Ais A, Guillemot F, Bounouader R, Cortot A, Romond C (1998). Cell-free whey from milk fermented with Bifidobacterium breve C50 used to modify the colonic microflora of healthy subjects. J Dairy Sci.

[103] Singh RK, Chang HW, Yan D, Lee KM, Ucmak D, Wong K, Abrouk M, Farahnik B, Nakamura M, Zhu TH, Bhutani T, Liao W (2017). Influence of diet on the gut microbiome and implications for human health. J Transl Med.

[104] Eid N, Enani S, Walton G, Corona G, Costabile A, Gibson G, Rowland I, Spencer JPE (2014). The impact of date palm fruits and their component polyphenols, on gut microbial ecology, bacterial metabolites and colon cancer cell proliferation. J Nutr Sci.

[105] Parvin S, Easmin D, Sheikh A, Biswas M, SCD S, MGS J (2015). Nutritional analysis of date fruits (*Phoenix dactylifera* L.) in perspective of Bangladesh. Am J Life Sci.

[106] Costabile A, Klinder A, Fava F, Napolitano A, Fogliano V, Leonard C, Gibson GR, Tuohy KM (2008). Whole-grain wheat breakfast cereal has a prebiotic effect on the human gut microbiota: a double-blind, placebo-controlled, crossover study. Br J Nutr.

[107] Carvalho-Wells AL, Helmolz K, Nodet C, Molzer C, Leonard C, McKevith B, Thielecke F, Jackson KG, Tuohy KM (2010). Determination of the in vivo prebiotic potential of a maize-based whole grain breakfast cereal: a human feeding study. Br J Nutr.

[108] Wu GD, Chen J, Hoffmann C, Bittinger K, Chen YY, Keilbaugh SA, Bewtra M, Knights D, Walters WA, Knight R, Sinha R, Gilroy E, Gupta K, Baldassano R, Nessel L, Li H, Bushman FD, Lewis JD (2011). Linking long-term dietary patterns with gut microbial enterotypes. Science..

[109] Reddy BS, Weisburger JH, Wynder EL (1975). Effects of high risk and low risk diets for colon carcinogenesis on fecal microflora and steroids in man. J Nutr.

[110] Drasar BS, Crowther JS, Goddard P, Hawksworth G, Hill MJ, Peach S, Williams REO, Renwich A (1973). The relation between diet and the gut microflora in man. Proc Nutr Soc.

[111] McFarland LV (2007). Meta-analysis of probiotics for the prevention of traveler's diarrhea. Travel Med Infect Dis.

[112] Pandey KR, Naik SR, Vakil BV (2015). Probiotics, prebiotics and synbiotics- a review. J Food Sci Technol.

[113] Fukuda S, Toh H, Hase K, Oshima K, Nakanishi Y, Yoshimura K, Tobe T, Clarke JM, Topping DL, Suzuki T, Taylor TD, Itoh K, Kikuchi J, Morita H, Hattori M, Ohno H (2011). Bifidobacteria can protect from enteropathogenic infection through production of acetate. Nature..

[114] Pinto-Sanchez MI, Hall GB, Ghajar K, Nardelli A, Bolino C, Lau JT, Martin FP, Cominetti O, Welsh C, Rieder A, Traynor J, Gregory C, de Palma G, Pigrau M, Ford AC, Macri J, Berger B, Bergonzelli G, Surette MG, Collins SM, Moayyedi P, Bercik P (2017). Probiotic Bifidobacterium longum NCC3001 reduces depression scores and alters brain activity: a pilot study in patients with irritable bowel syndrome. Gastroenterology..

[115] Bercik P, Park AJ, Sinclair D, Khoshdel A, Lu J, Huang X, Deng Y, Blennerhassett PA, Fahnestock M, Moine D, Berger B, Huizinga JD, Kunze W, McLean PG, Bergonzelli GE, Collins SM, Verdu EF (2011). The anxiolytic effect of Bifidobacterium longum NCC3001 involves vagal pathways for gut-brain communication. Neurogastroenterol Motil.

[116] Messaoudi M, Lalonde R, Violle N, Javelot H, Desor D, Nejdi A, Bisson JF, Rougeot C, Pichelin M, Cazaubiel M, Cazaubiel JM (2011). Assessment of psychotropic-like properties of a probiotic formulation (Lactobacillus helveticus R0052 and Bifidobacterium longum R0175) in rats and human subjects. Br J Nutr.

[117] Desbonnet L, Garrett L, Clarke G, Kiely B, Cryan JF, Dinan TG (2010). Effects of the probiotic Bifidobacterium infantis in the maternal separation model of depression. Neuroscience..

[118] Saavedra JM, Bauman NA, Oung I, Perman JA, Yolken RH (1994). Feeding of Bifidobacterium bifidum and Streptococcus thermophilus to infants in hospital for prevention of diarrhoea and shedding of rotavirus. Lancet..

[119] Bernet MF, Brassart D, Neeser JR, Servin AL (1993). Adhesion of human bifidobacterial strains to cultured human intestinal epithelial cells and inhibition of enteropathogen-cell interactions. Appl Environ Microbiol.

[120] Sekine K, Toida T, Saito M, Kuboyama M, Kawashima T, Hashimoto Y (1985). A new morphologically characterized cell wall preparation (whole peptidoglycan) from Bifidobacterium infantis with a higher efficacy on the regression of an established tumor in mice. Cancer Res.

[121] Rowland IR, Rumney CJ, Coutts JT, Lievense LC (1998). Effect of Bifidobacterium longum and inulin on gut bacterial metabolism and carcinogen-induced aberrant crypt foci in rats. Carcinogenesis..

[122] Le Leu RK, Hu Y, Brown IL, Woodman RJ, Young GP (2010). Synbiotic intervention of Bifidobacterium lactis and resistant starch protects against colorectal cancer development in rats. Carcinogenesis..

[123] Tavan E, Cayuela C, Antoine JM, Cassand P (2002). Antimutagenic activities of various lactic acid bacteria against food mutagens: heterocyclic amines. J Dairy Res..

[124] Pool-Zobel BL, Neudecker C, Domizlaff I, Ji S, Schillinger U, Rumney C, Moretti M, Vilarini I, Scassellati-Sforzolini R, Rowland I (1996). Lactobacillus- and bifidobacterium-mediated antigenotoxicity in the colon of rats. Nutr Cancer.

[125] Chenoll E, Rivero M, Codoner FM, Martinez-Blanch JF, Ramon D, Genoves S, et al. Complete genome sequence of Bifidobacterium longum subsp. infantis Strain CECT 7210, a probiotic strain active against rotavirus infections. Genome Announc. 2015;3(2). 10.1128/genomeA.00105-15. 10.1128/genomeA.00105-15 PMC4384477 25838473

[126] Patole SK, Rao SC, Keil AD, Nathan EA, Doherty DA, Simmer KN (2016). Benefits of Bifidobacterium breve M-16V supplementation in preterm neonates - a retrospective cohort study. PLoS One.

[127] Venturi A, Gionchetti P, Rizzello F, Johansson R, Zucconi E, Brigidi P, Matteuzzi D, Campieri M (1999). Impact on the composition of the faecal flora by a new probiotic preparation: preliminary data on maintenance treatment of patients with ulcerative colitis. Aliment Pharmacol Ther.

[128] Gionchetti P, Rizzello F, Venturi A, Campieri M (2000). Probiotics in infective diarrhoea and inflammatory bowel diseases. J Gastroenterol Hepatol.

[129] Marteau P, Cuillerier E, Meance S, Gerhardt MF, Myara A, Bouvier M, Bouley C, Tondu F, Bommelaer G, Grimaud JC (2002). Bifidobacterium animalis strain DN-173 010 shortens the colonic transit time in healthy women: a double-blind, randomized, controlled study. Aliment Pharmacol Ther.

[130] Andrade S, Borges N (2009). Effect of fermented milk containing Lactobacillus acidophilus and Bifidobacterium longum on plasma lipids of women with normal or moderately elevated cholesterol. J Dairy Res..

[131] Guyonnet D, Chassany O, Ducrotte P, Picard C, Mouret M, Mercier CH (2007). Effect of a fermented milk containing Bifidobacterium animalis DN-173 010 on the health-related quality of life and symptoms in irritable bowel syndrome in adults in primary care: a multicentre, randomized, double-blind, controlled trial. Aliment Pharmacol Ther.

[132] Yang YX, He M, Hu G, Wei J, Pages P, Yang XH, Bourdu-Naturel S (2008). Effect of a fermented milk containing Bifidobacterium lactis DN-173010 on Chinese constipated women. World J Gastroenterol.

[133] Kleessen B, Sykura B, Zunft HJ, Blaut M (1997). Effects of inulin and lactose on fecal microflora, microbial activity, and bowel habit in elderly constipated persons. Am J Clin Nutr.

[134] Duffy LC, Zielezny MA, Riepenhoff-Talty M, Dryja D, Sayahtaheri-Altaie S, Griffiths E, Ruffin D, Barrett H, Ogra PL (1994). Reduction of virus shedding by B. bifidum in experimentally induced MRV infection. Statistical application for ELISA. Dig Dis Sci.

[135] Duffy LC, Zielezny MA, Riepenhoff-Talty M, Dryja D, Sayahtaheri-Altaie S, Griffiths E, Ruffin D, Barrett H, Rossman J, Ogra PL (1993). Effectiveness of Bifidobacterium bifidum in experimentally induced MRV infection: dietary implications in formulas for newborns. Endocr Regul.

[136] Perdigon G, Alvarez S, Rachid M, Aguero G, Gobbato N (1995). Immune system stimulation by probiotics. J Dairy Sci.

[137] Picard C, Fioramonti J, Francois A, Robinson T, Neant F, Matuchansky C (2005). Review article: bifidobacteria as probiotic agents -- physiological effects and clinical benefits. Aliment Pharmacol Ther.

[138] Gueimonde M, Margolles A, de los Reyes-Gavilan CG, Salminen S (2007). Competitive exclusion of enteropathogens from human intestinal mucus by Bifidobacterium strains with acquired resistance to bile--a preliminary study. Int J Food Microbiol.

[139] Schroeder BO, Birchenough GMH, Stahlman M, Arike L, Johansson MEV, Hansson GC (2018). Bifidobacteria or fiber protects against diet-induced microbiota-mediated colonic mucus deterioration. Cell Host Microbe.

[140] Gomi A, Harima-Mizusawa N, Shibahara-Sone H, Kano M, Miyazaki K, Ishikawa F (2013). Effect of Bifidobacterium bifidum BF-1 on gastric protection and mucin production in an acute gastric injury rat model. J Dairy Sci.

[141] Javed NH, Alsahly MB, Khubchandani J (2016). Oral feeding of probiotic Bifidobacterium infantis: colonic morphological changes in rat model of TNBS-induced colitis. Scientifica (Cairo).

[142] Kawahara T, Makizaki Y, Oikawa Y, Tanaka Y, Maeda A, Shimakawa M, Komoto S, Moriguchi K, Ohno H, Taniguchi K (2017). Oral administration of Bifidobacterium bifidum G9-1 alleviates rotavirus gastroenteritis through regulation of intestinal homeostasis by inducing mucosal protective factors. PLoS One.

[143] Khailova L, Dvorak K, Arganbright KM, Halpern MD, Kinouchi T, Yajima M, Dvorak B (2009). Bifidobacterium bifidum improves intestinal integrity in a rat model of necrotizing enterocolitis. Am J Physiol Gastrointest Liver Physiol.

