# Supplementary material for: The metabolic profile of Bifidobacterium dentium reflects its status as a human gut commensal
Source: BMC Microbiol. 2021 May 24;21:154. doi: 10.1186/s12866-021-02166-6 (PMC8145834; doi:10.1186/s12866-021-02166-6)
Supplement: Supplementary file 1 — Additional file 1. [file 12866_2021_2166_MOESM1_ESM.docx]

**Supplemental Table 1:** Statistics from growth curves at time point, 8.3 hours**.** Significant p values are denoted as follows: *P<0.05, **P<0.01, ***P<0.001, ****P<0.0001

|  | 8.3 hrs |  |  |
| --- | --- | --- | --- |
| **Comparison** | **95.00% CI of diff.** | **Significant?** | **P Value** |
| Negative vs. D-Galactose | -0.7584 to -0.09959 | ** | 0.0022 |
| Negative vs. D-Mannose | -0.8634 to -0.2046 | **** | <0.0001 |
| Negative vs. Glucuronamide | -0.4424 to 0.2164 | ns | 0.9982 |
| Negative vs. beta-D-Allose | -0.2814 to 0.3774 | ns | 0.9994 |
| Negative vs. alpha-D-Glucose | -0.8554 to -0.1966 | **** | <0.0001 |
| Negative vs. L-Glucose | -0.2804 to 0.3784 | ns | 0.9994 |
| Negative vs. Sedoheptulosan | -0.7294 to -0.07059 | ** | 0.0061 |
| Negative vs. D-Fructose | -0.4394 to 0.2194 | ns | 0.9983 |
| Negative vs. D-Psicose | -0.2894 to 0.3694 | ns | 0.9995 |
| Negative vs. L-Sorbose | -0.2794 to 0.3794 | ns | 0.9994 |
| Negative vs. D-Tagatose | -0.4994 to 0.1594 | ns | 0.8652 |
| Negative vs. L-Arabanose | -0.5194 to 0.1394 | ns | 0.7253 |
| Negative vs. D-Xylose | -0.6594 to -0.0005865 | * | 0.0492 |
| Negative vs. D-Ribose | -0.4594 to 0.1994 | ns | 0.9846 |
| Negative vs. L-Lyxose | -0.2894 to 0.3694 | ns | 0.9995 |
| Negative vs. D-Arabinose | -0.2794 to 0.3794 | ns | 0.9994 |
| Negative vs. D-Sorbitol | -0.4294 to 0.2294 | ns | 0.9985 |
| Negative vs. D-Mannitol | -0.7794 to -0.1206 | ** | 0.0011 |
| Negative vs. Adonitol | -0.3494 to 0.3094 | ns | 0.9997 |
| Negative vs. D-Arabitol | -0.2794 to 0.3794 | ns | 0.9994 |
| Negative vs. L-Arabitol | -0.2794 to 0.3794 | ns | 0.9994 |
| Negative vs. i-Erythritol | -0.2794 to 0.3794 | ns | 0.9994 |
| Negative vs. Lactitol | -0.2794 to 0.3794 | ns | 0.9994 |
| Negative vs. Maltitol | -0.2894 to 0.3694 | ns | 0.9995 |
| Negative vs. Xylitol | -0.2794 to 0.3794 | ns | 0.9994 |
| Negative vs. Dulcitol | -0.3594 to 0.2994 | ns | 0.9996 |
| Negative vs. alpha-D-Lactose | -0.3594 to 0.2994 | ns | 0.9996 |
| Negative vs. D-Cellobiose | -0.3594 to 0.2994 | ns | 0.9996 |
| Negative vs. D-Melibiose | -0.7194 to -0.06059 | ** | 0.0084 |
| Negative vs. D-Trehalose | -0.3694 to 0.2894 | ns | 0.9995 |
| Negative vs. Gentiobiose | -0.7994 to -0.1406 | *** | 0.0005 |
| Negative vs. Lactulose | -0.3594 to 0.2994 | ns | 0.9996 |
| Negative vs. Maltose | -0.9494 to -0.2906 | **** | <0.0001 |
| Negative vs. Palatinose | -0.2894 to 0.3694 | ns | 0.9995 |
| Negative vs. Sucrose | -1.049 to -0.3906 | **** | <0.0001 |
| Negative vs. Turanose | -0.8294 to -0.1706 | *** | 0.0002 |
| Negative vs. D-Melezitose | -0.2794 to 0.3794 | ns | 0.9994 |
| Negative vs. D-Raffinose | -0.8694 to -0.2106 | **** | <0.0001 |
| Negative vs. Maltotriose | -0.9094 to -0.2506 | **** | <0.0001 |
| Negative vs. Stachyose | -0.8794 to -0.2206 | **** | <0.0001 |
| Negative vs. L-Fucose | -0.4194 to 0.2394 | ns | 0.9987 |
| Negative vs. D-Fucose | -0.2794 to 0.3794 | ns | 0.9994 |
| Negative vs. 2-Deoxy-D Ribose | -0.2794 to 0.3794 | ns | 0.9994 |
| Negative vs. L-Rhamnose | -0.3994 to 0.2594 | ns | 0.9991 |
| Negative vs. N-Acetyl-D Glucosaminitol | -0.2794 to 0.3794 | ns | 0.9994 |
| Negative vs. N-Acetyl-DGalactosamine | -0.2894 to 0.3694 | ns | 0.9995 |
| Negative vs. N-Acetyl-D-Glucosamine | -0.4194 to 0.2394 | ns | 0.9987 |
| Negative vs. D-Glucosamine | -0.2894 to 0.3694 | ns | 0.9995 |
| Negative vs. N-AcetylNeuraminic Acid | -0.2794 to 0.3794 | ns | 0.9994 |
| Negative vs. 2-Hydroxy Benzoic Acid | -0.2114 to 0.2514 | ns | 0.9997 |
| Negative vs. 4-Hydroxy Benzoic Acid | -0.2114 to 0.2514 | ns | 0.9997 |
| Negative vs. 5-Keto-D Gluconic Acid | -0.2114 to 0.2514 | ns | 0.9997 |
| Negative vs. Acetic Acid | -0.3214 to 0.1414 | ns | 0.9858 |
| Negative vs. Acetoacetic Acid | -0.3014 to 0.1614 | ns | 0.9985 |
| Negative vs. a-Keto-Valeric Acid | -0.2114 to 0.2514 | ns | 0.9997 |
| Negative vs. alpha-Hydroxy Butyric Acid | -0.2814 to 0.1814 | ns | 0.9991 |
| Negative vs. Column I | -0.3014 to 0.1614 | ns | 0.9985 |
| Negative vs. alpha-Keto-Butyric Acid | -0.3114 to 0.1514 | ns | 0.9982 |
| Negative vs. alpha-Keto-Glutaric Acid | -0.3214 to 0.1414 | ns | 0.9858 |
| Negative vs. beta-Hydroxy Butyric Acid | -0.2114 to 0.2514 | ns | 0.9997 |
| Negative vs. beta-Methyl-DGlucuronic Acid | -0.2114 to 0.2514 | ns | 0.9997 |
| Negative vs. Bromo Succinic Acid | -0.2914 to 0.1714 | ns | 0.9988 |
| Negative vs. Butyric Acid | -0.2114 to 0.2514 | ns | 0.9997 |
| Negative vs. Capric Acid | -0.2614 to 0.2014 | ns | 0.9995 |
| Negative vs. Caproic Acid | -0.2114 to 0.2514 | ns | 0.9997 |
| Negative vs. Citraconic Acid | -0.2114 to 0.2514 | ns | 0.9997 |
| Negative vs. Citramalic Acid | -0.2114 to 0.2514 | ns | 0.9997 |
| Negative vs. Citric Acid | -0.3314 to 0.1314 | ns | 0.9811 |
| Negative vs. D,L-Malic Acid | -0.3414 to 0.1214 | ns | 0.948 |
| Negative vs. delta-Amino Valeric Acid | -0.2114 to 0.2514 | ns | 0.9997 |
| Negative vs. Column W | -0.3514 to 0.1114 | ns | 0.881 |
| Negative vs. D-Galacturonic Acid | -0.2614 to 0.2014 | ns | 0.9995 |
| Negative vs. D-Gluconic Acid | -0.5014 to -0.03863 | ** | 0.0097 |
| Negative vs. D-Glucosaminic Acid | -0.3414 to 0.1214 | ns | 0.948 |
| Negative vs. D-Glucuronic Acid | -0.3514 to 0.1114 | ns | 0.881 |
| Negative vs. D-Lactic Acid Methyl Ester | -0.2214 to 0.2414 | ns | 0.9999 |
| Negative vs. D-Malic Acid | -0.2714 to 0.1914 | ns | 0.9993 |
| Negative vs. D-Saccharic Acid | -0.3414 to 0.1214 | ns | 0.948 |
| Negative vs. D-Tartaric Acid | -0.2114 to 0.2514 | ns | 0.9997 |
| Negative vs. Formic Acid | -0.2914 to 0.1714 | ns | 0.9988 |
| Negative vs. Fumaric Acid | -0.3114 to 0.1514 | ns | 0.9982 |
| Negative vs. gamma-Amino Butyric Acid | -0.2114 to 0.2514 | ns | 0.9997 |
| Negative vs. gamma-Hydroxy Butyric Acid | -0.2114 to 0.2514 | ns | 0.9997 |
| Negative vs. Glycolic Acid | -0.2814 to 0.1814 | ns | 0.9991 |
| Negative vs. Glyoxylic Acid | -0.2214 to 0.2414 | ns | 0.9999 |
| Negative vs. Itaconic Acid | -0.2114 to 0.2514 | ns | 0.9997 |
| Negative vs. Column AM | -0.2814 to 0.1814 | ns | 0.9991 |
| Negative vs. L-Lactic Acid | -0.3114 to 0.1514 | ns | 0.9982 |
| Negative vs. L-Malic Acid | -0.2614 to 0.2014 | ns | 0.9995 |
| Negative vs. L-Tartaric Acid | -0.2114 to 0.2514 | ns | 0.9997 |
| Negative vs. Malonic Acid | -0.2114 to 0.2514 | ns | 0.9997 |
| Negative vs. Melibionic Acid | -0.2114 to 0.2514 | ns | 0.9997 |
| Negative vs. Methyl Pyruvate | -0.2814 to 0.1814 | ns | 0.9991 |
| Negative vs. m-Hydroxy Phenyl Acetic Acid | -0.3814 to 0.08137 | ns | 0.5605 |
| Negative vs. m-Tartaric Acid | -0.3314 to 0.1314 | ns | 0.9811 |
| Negative vs. Mucic Acid | -0.3014 to 0.1614 | ns | 0.9985 |
| Negative vs. N-Acetyl-LGlutamic Acid | -0.2114 to 0.2514 | ns | 0.9997 |
| Negative vs. Oxalic Acid | -0.2114 to 0.2514 | ns | 0.9997 |
| Negative vs. Oxalomalic Acid | -0.2114 to 0.2514 | ns | 0.9997 |
| Negative vs. p-Hydroxy Phenyl Acetic Acid | -0.4014 to 0.06137 | ns | 0.3562 |
| Negative vs. Propionic Acid | -0.2914 to 0.1714 | ns | 0.9988 |
| Negative vs. Pyruvic Acid | -0.3014 to 0.1614 | ns | 0.9985 |
| Negative vs. Quinic Acid | -0.2114 to 0.2514 | ns | 0.9997 |
| Negative vs. Sebacic Acid | -0.2114 to 0.2514 | ns | 0.9997 |
| Negative vs. Sorbic Acid | -0.2114 to 0.2514 | ns | 0.9997 |
| Negative vs. Succinamic Acid | -0.2114 to 0.2514 | ns | 0.9997 |
| Negative vs. Succinic Acid | -0.3314 to 0.1314 | ns | 0.9811 |
| Negative vs. Tricarballylic Acid | -0.3414 to 0.1214 | ns | 0.948 |
| Negative vs. D-Alanine | -0.4137 to 0.2737 | ns | 0.9991 |
| Negative vs. D-Aspartic Acid | -0.4537 to 0.2337 | ns | 0.9937 |
| Negative vs. D-Serine | -0.4437 to 0.2437 | ns | 0.9947 |
| Negative vs. D-Threonine | -0.4337 to 0.2537 | ns | 0.9988 |
| Negative vs. Glycine | -0.3237 to 0.3637 | ns | 0.9998 |
| Negative vs. L-Alanine | -0.4237 to 0.2637 | ns | 0.999 |
| Negative vs. L-Arginine | -0.3237 to 0.3637 | ns | 0.9998 |
| Negative vs. L-Asparagine | -0.4437 to 0.2437 | ns | 0.9947 |
| Negative vs. L-Aspartic Acid | -0.4437 to 0.2437 | ns | 0.9947 |
| Negative vs. L-Glutamic Acid | -0.4237 to 0.2637 | ns | 0.999 |
| Negative vs. L-Glutamine | -0.4537 to 0.2337 | ns | 0.9937 |
| Negative vs. L-Histidine | -0.3237 to 0.3637 | ns | 0.9998 |
| Negative vs. L-Isoleucine | -0.3237 to 0.3637 | ns | 0.9998 |
| Negative vs. L-Leucine | -0.3337 to 0.3537 | ns | 0.9999 |
| Negative vs. L-Lysine | -0.3237 to 0.3637 | ns | 0.9998 |
| Negative vs. L-Methionine | -0.3237 to 0.3637 | ns | 0.9998 |
| Negative vs. L-Ornithine | -0.3337 to 0.3537 | ns | 0.9999 |
| Negative vs. L-Phenylalanine | -0.3237 to 0.3637 | ns | 0.9998 |
| Negative vs. L-Proline | -0.7237 to -0.03635 | * | 0.0206 |
| Negative vs. L-Serine | -0.4437 to 0.2437 | ns | 0.9947 |
| Negative vs. L-Threonine | -0.4337 to 0.2537 | ns | 0.9988 |
| Negative vs. L-Valine | -0.3237 to 0.3637 | ns | 0.9998 |
| Negative vs. Tyramine | -0.4337 to 0.2537 | ns | 0.9988 |
| Negative vs. Glycyl-L-Aspartic Acid | -0.4537 to 0.2337 | ns | 0.9937 |
| Negative vs. Glycyl-LGlutamic Acid | -0.4437 to 0.2437 | ns | 0.9947 |
| Negative vs. Glycyl-L-Proline | -0.4537 to 0.2337 | ns | 0.9937 |
| Negative vs. D,L-Octopamine | -0.005093 to 0.02509 | ns | 0.1982 |
| Negative vs. Sec-Butylamine | 0.004907 to 0.03509 | * | 0.013 |
| Negative vs. L-Alaninamide | -0.09386 to 0.1139 | ns | 0.9965 |
| Negative vs. L-Alanyl-Glycine | -0.1739 to 0.03386 | ns | 0.2419 |
| Negative vs. L-Homoserine | -0.08386 to 0.1239 | ns | 0.9557 |
| Negative vs. L-Pyroglutamic Acid | -0.08386 to 0.1239 | ns | 0.9557 |
| Negative vs. D,L-Carnitine | 0.009328 to 0.03067 | ** | 0.0016 |
| Negative vs. Putrescine | 0.009328 to 0.03067 | ** | 0.0016 |
| Negative vs. m-Inositol | -0.2220 to 0.08499 | ns | 0.9512 |
| Negative vs. Mono Methyl Succinate | -0.1735 to 0.1335 | ns | 0.9995 |
| Negative vs. Gelatin | -0.1075 to 0.1995 | ns | 0.9985 |
| Negative vs. 1,2-Propanediol | -0.2205 to 0.08649 | ns | 0.9613 |
| Negative vs. 2,3-Butanediol | -0.1250 to 0.1820 | ns | 0.9992 |
| Negative vs. 3-Hydroxy 2-Butanone | -0.1135 to 0.1935 | ns | 0.9988 |
| Negative vs. Chondroitin Sulfate C | -0.1080 to 0.1990 | ns | 0.9985 |
| Negative vs. Amygdalin | -0.4010 to -0.09401 | **** | <0.0001 |
| Negative vs. N-Acetyl-beta-D Mannosamine | -0.1890 to 0.1180 | ns | 0.999 |
| Negative vs. Acetamide | -0.1195 to 0.1875 | ns | 0.999 |
| Negative vs. 2-Aminoethanol | -0.1445 to 0.1625 | ns | 0.9998 |
| Negative vs. Phenylethylamine | -0.1610 to 0.1460 | ns | 0.9998 |
| Negative vs. Glycerol | -0.2525 to 0.05449 | ns | 0.5278 |
| Negative vs. D-Ribono-1,4-Lactone | -0.1060 to 0.2010 | ns | 0.9985 |
| Negative vs. Dihydroxy Acetone | -0.1095 to 0.1975 | ns | 0.9986 |
| Negative vs. alpha-Cyclodextrin | -0.1050 to 0.2020 | ns | 0.9984 |
| Negative vs. beta-Cyclodextrin | -0.1055 to 0.2015 | ns | 0.9985 |
| Negative vs. gamma-Cyclodextrin | -0.1085 to 0.1985 | ns | 0.9986 |
| Negative vs. Dextrin | -0.1390 to 0.1680 | ns | 0.9996 |
| Negative vs. Glycogen | -0.1320 to 0.1750 | ns | 0.9994 |
| Negative vs. Inulin | -0.1045 to 0.2025 | ns | 0.9984 |
| Negative vs. Laminarin | -0.1065 to 0.2005 | ns | 0.9985 |
| Negative vs. Mannan | -0.1095 to 0.1975 | ns | 0.9986 |
| Negative vs. Pectin | -0.1375 to 0.1695 | ns | 0.9996 |
| Negative vs. Tween 20 | -0.1650 to 0.1420 | ns | 0.9997 |
| Negative vs. Tween 40 | -0.1680 to 0.1390 | ns | 0.9996 |
| Negative vs. Tween 80 | -0.1535 to 0.1535 | ns | >0.9999 |
| Negative vs. 2-Deoxy Adenosine | -0.2225 to 0.08449 | ns | 0.9475 |
| Negative vs. Adenosine | -0.2205 to 0.08649 | ns | 0.9613 |
| Negative vs. Inosine | -0.2915 to 0.01549 | ns | 0.112 |
| Negative vs. Thymidine | -0.1855 to 0.1215 | ns | 0.9991 |
| Negative vs. Uridine | -0.2110 to 0.09599 | ns | 0.9859 |
| Negative vs. alpha-Methyl-D-Galactoside | -0.2085 to 0.09849 | ns | 0.9927 |
| Negative vs. alpha-Methyl-DMannoside | -0.1050 to 0.2020 | ns | 0.9984 |
| Negative vs. a-Methyl-D Glucoside | -0.1110 to 0.1960 | ns | 0.9987 |
| Negative vs. beta-Methyl-D Glucoside | -0.1915 to 0.1155 | ns | 0.9989 |
| Negative vs. beta-Methyl-DGalactoside | -0.1075 to 0.1995 | ns | 0.9985 |
| Negative vs. beta-Methyl-DXyloside | -0.1045 to 0.2025 | ns | 0.9984 |
| Negative vs. Arbutin | -0.3325 to -0.02551 | * | 0.0107 |
| Negative vs. Salicin | -0.3335 to -0.02651 | * | 0.01 |

**Supplemental Table 2:** Statistics from growth curves at time point, 16 hours**.** Significant p values are denoted as follows: *P<0.05, **P<0.01, ***P<0.001, ****P<0.0001

|  | 16 hrs |  |  |
| --- | --- | --- | --- |
| **Comparison** | **95.00% CI of diff.** | **Significant?** | **P Value** |
| Negative vs. D-Galactose | -0.4741 to 0.1441 | ns | 0.8281 |
| Negative vs. D-Mannose | -0.8481 to -0.2299 | **** | <0.0001 |
| Negative vs. Glucuronamide | -0.3261 to 0.2921 | ns | 0.9998 |
| Negative vs. beta-D-Allose | -0.2311 to 0.3871 | ns | 0.9988 |
| Negative vs. alpha-D-Glucose | -0.9221 to -0.3039 | **** | <0.0001 |
| Negative vs. L-Glucose | -0.2301 to 0.3881 | ns | 0.9988 |
| Negative vs. Sedoheptulosan | -0.6891 to -0.07092 | ** | 0.0052 |
| Negative vs. D-Fructose | -0.6091 to 0.009078 | ns | 0.0643 |
| Negative vs. D-Psicose | -0.2291 to 0.3891 | ns | 0.9988 |
| Negative vs. L-Sorbose | -0.2291 to 0.3891 | ns | 0.9988 |
| Negative vs. D-Tagatose | -0.3391 to 0.2791 | ns | 0.9996 |
| Negative vs. L-Arabanose | -0.4391 to 0.1791 | ns | 0.9816 |
| Negative vs. D-Xylose | -0.5391 to 0.07908 | ns | 0.3262 |
| Negative vs. D-Ribose | -0.3691 to 0.2491 | ns | 0.9992 |
| Negative vs. L-Lyxose | -0.2291 to 0.3891 | ns | 0.9988 |
| Negative vs. D-Arabinose | -0.2291 to 0.3891 | ns | 0.9988 |
| Negative vs. D-Sorbitol | -0.3091 to 0.3091 | ns | >0.9999 |
| Negative vs. D-Mannitol | -0.6491 to -0.03092 | * | 0.0198 |
| Negative vs. Adonitol | -0.2691 to 0.3491 | ns | 0.9995 |
| Negative vs. D-Arabitol | -0.2291 to 0.3891 | ns | 0.9988 |
| Negative vs. L-Arabitol | -0.2391 to 0.3791 | ns | 0.999 |
| Negative vs. i-Erythritol | -0.2291 to 0.3891 | ns | 0.9988 |
| Negative vs. Lactitol | -0.2291 to 0.3891 | ns | 0.9988 |
| Negative vs. Maltitol | -0.2691 to 0.3491 | ns | 0.9995 |
| Negative vs. Xylitol | -0.2391 to 0.3791 | ns | 0.999 |
| Negative vs. Dulcitol | -0.3091 to 0.3091 | ns | >0.9999 |
| Negative vs. alpha-D-Lactose | -0.2791 to 0.3391 | ns | 0.9996 |
| Negative vs. D-Cellobiose | -0.2691 to 0.3491 | ns | 0.9995 |
| Negative vs. D-Melibiose | -0.5191 to 0.09908 | ns | 0.4653 |
| Negative vs. D-Trehalose | -0.2991 to 0.3191 | ns | 0.9999 |
| Negative vs. Gentiobiose | -0.8191 to -0.2009 | **** | <0.0001 |
| Negative vs. Lactulose | -0.2691 to 0.3491 | ns | 0.9995 |
| Negative vs. Maltose | -0.7591 to -0.1409 | *** | 0.0004 |
| Negative vs. Palatinose | -0.2491 to 0.3691 | ns | 0.9992 |
| Negative vs. Sucrose | -0.8091 to -0.1909 | **** | <0.0001 |
| Negative vs. Turanose | -0.8791 to -0.2609 | **** | <0.0001 |
| Negative vs. D-Melezitose | -0.2291 to 0.3891 | ns | 0.9988 |
| Negative vs. D-Raffinose | -0.7291 to -0.1109 | ** | 0.0012 |
| Negative vs. Maltotriose | -0.8391 to -0.2209 | **** | <0.0001 |
| Negative vs. Stachyose | -0.7891 to -0.1709 | *** | 0.0001 |
| Negative vs. L-Fucose | -0.3091 to 0.3091 | ns | >0.9999 |
| Negative vs. D-Fucose | -0.2291 to 0.3891 | ns | 0.9988 |
| Negative vs. 2-Deoxy-D Ribose | -0.2391 to 0.3791 | ns | 0.999 |
| Negative vs. L-Rhamnose | -0.3091 to 0.3091 | ns | >0.9999 |
| Negative vs. N-Acetyl-D Glucosaminitol | -0.2291 to 0.3891 | ns | 0.9988 |
| Negative vs. N-Acetyl-DGalactosamine | -0.2291 to 0.3891 | ns | 0.9988 |
| Negative vs. N-Acetyl-D-Glucosamine | -0.3091 to 0.3091 | ns | >0.9999 |
| Negative vs. D-Glucosamine | -0.2391 to 0.3791 | ns | 0.999 |
| Negative vs. N-AcetylNeuraminic Acid | -0.2291 to 0.3891 | ns | 0.9988 |
| Negative vs. 2-Hydroxy Benzoic Acid | -0.1374 to 0.1374 | ns | >0.9999 |
| Negative vs. 4-Hydroxy Benzoic Acid | -0.1274 to 0.1474 | ns | 0.9997 |
| Negative vs. 5-Keto-D Gluconic Acid | -0.1274 to 0.1474 | ns | 0.9997 |
| Negative vs. Acetic Acid | -0.1974 to 0.07743 | ns | 0.9804 |
| Negative vs. Acetoacetic Acid | -0.1674 to 0.1074 | ns | 0.999 |
| Negative vs. a-Keto-Valeric Acid | -0.1274 to 0.1474 | ns | 0.9997 |
| Negative vs. alpha-Hydroxy Butyric Acid | -0.1774 to 0.09743 | ns | 0.9986 |
| Negative vs. Column I | -0.1874 to 0.08743 | ns | 0.998 |
| Negative vs. alpha-Keto-Butyric Acid | -0.1874 to 0.08743 | ns | 0.998 |
| Negative vs. alpha-Keto-Glutaric Acid | -0.2074 to 0.06743 | ns | 0.8984 |
| Negative vs. beta-Hydroxy Butyric Acid | -0.1274 to 0.1474 | ns | 0.9997 |
| Negative vs. beta-Methyl-DGlucuronic Acid | -0.1274 to 0.1474 | ns | 0.9997 |
| Negative vs. Bromo Succinic Acid | -0.1874 to 0.08743 | ns | 0.998 |
| Negative vs. Butyric Acid | -0.1274 to 0.1474 | ns | 0.9997 |
| Negative vs. Capric Acid | -0.1374 to 0.1374 | ns | >0.9999 |
| Negative vs. Caproic Acid | -0.1274 to 0.1474 | ns | 0.9997 |
| Negative vs. Citraconic Acid | -0.1274 to 0.1474 | ns | 0.9997 |
| Negative vs. Citramalic Acid | -0.1274 to 0.1474 | ns | 0.9997 |
| Negative vs. Citric Acid | -0.2174 to 0.05743 | ns | 0.7353 |
| Negative vs. D,L-Malic Acid | -0.2074 to 0.06743 | ns | 0.8984 |
| Negative vs. delta-Amino Valeric Acid | -0.1274 to 0.1474 | ns | 0.9997 |
| Negative vs. Column W | -0.2074 to 0.06743 | ns | 0.8984 |
| Negative vs. D-Galacturonic Acid | -0.1474 to 0.1274 | ns | 0.9997 |
| Negative vs. D-Gluconic Acid | -0.3574 to -0.08257 | **** | <0.0001 |
| Negative vs. D-Glucosaminic Acid | -0.2074 to 0.06743 | ns | 0.8984 |
| Negative vs. D-Glucuronic Acid | -0.2074 to 0.06743 | ns | 0.8984 |
| Negative vs. D-Lactic Acid Methyl Ester | -0.1274 to 0.1474 | ns | 0.9997 |
| Negative vs. D-Malic Acid | -0.1574 to 0.1174 | ns | 0.9994 |
| Negative vs. D-Saccharic Acid | -0.2074 to 0.06743 | ns | 0.8984 |
| Negative vs. D-Tartaric Acid | -0.1274 to 0.1474 | ns | 0.9997 |
| Negative vs. Formic Acid | -0.1874 to 0.08743 | ns | 0.998 |
| Negative vs. Fumaric Acid | -0.1874 to 0.08743 | ns | 0.998 |
| Negative vs. gamma-Amino Butyric Acid | -0.1274 to 0.1474 | ns | 0.9997 |
| Negative vs. gamma-Hydroxy Butyric Acid | -0.1274 to 0.1474 | ns | 0.9997 |
| Negative vs. Glycolic Acid | -0.1674 to 0.1074 | ns | 0.999 |
| Negative vs. Glyoxylic Acid | -0.1374 to 0.1374 | ns | >0.9999 |
| Negative vs. Itaconic Acid | -0.1274 to 0.1474 | ns | 0.9997 |
| Negative vs. Column AM | -0.1574 to 0.1174 | ns | 0.9994 |
| Negative vs. L-Lactic Acid | -0.1974 to 0.07743 | ns | 0.9804 |
| Negative vs. L-Malic Acid | -0.1474 to 0.1274 | ns | 0.9997 |
| Negative vs. L-Tartaric Acid | -0.1274 to 0.1474 | ns | 0.9997 |
| Negative vs. Malonic Acid | -0.1274 to 0.1474 | ns | 0.9997 |
| Negative vs. Melibionic Acid | -0.1274 to 0.1474 | ns | 0.9997 |
| Negative vs. Methyl Pyruvate | -0.1774 to 0.09743 | ns | 0.9986 |
| Negative vs. m-Hydroxy Phenyl Acetic Acid | -0.1874 to 0.08743 | ns | 0.998 |
| Negative vs. m-Tartaric Acid | -0.2074 to 0.06743 | ns | 0.8984 |
| Negative vs. Mucic Acid | -0.1974 to 0.07743 | ns | 0.9804 |
| Negative vs. N-Acetyl-LGlutamic Acid | -0.1274 to 0.1474 | ns | 0.9997 |
| Negative vs. Oxalic Acid | -0.1274 to 0.1474 | ns | 0.9997 |
| Negative vs. Oxalomalic Acid | -0.1274 to 0.1474 | ns | 0.9997 |
| Negative vs. p-Hydroxy Phenyl Acetic Acid | -0.1974 to 0.07743 | ns | 0.9804 |
| Negative vs. Propionic Acid | -0.1774 to 0.09743 | ns | 0.9986 |
| Negative vs. Pyruvic Acid | -0.1974 to 0.07743 | ns | 0.9804 |
| Negative vs. Quinic Acid | -0.1274 to 0.1474 | ns | 0.9997 |
| Negative vs. Sebacic Acid | -0.1274 to 0.1474 | ns | 0.9997 |
| Negative vs. Sorbic Acid | -0.1274 to 0.1474 | ns | 0.9997 |
| Negative vs. Succinamic Acid | -0.1274 to 0.1474 | ns | 0.9997 |
| Negative vs. Succinic Acid | -0.2074 to 0.06743 | ns | 0.8984 |
| Negative vs. Tricarballylic Acid | -0.1974 to 0.07743 | ns | 0.9804 |
| Negative vs. D-Alanine | -0.1996 to 0.09964 | ns | 0.9882 |
| Negative vs. D-Aspartic Acid | -0.2096 to 0.08964 | ns | 0.9619 |
| Negative vs. D-Serine | -0.1996 to 0.09964 | ns | 0.9882 |
| Negative vs. D-Threonine | -0.1996 to 0.09964 | ns | 0.9882 |
| Negative vs. Glycine | -0.1396 to 0.1596 | ns | 0.9997 |
| Negative vs. L-Alanine | -0.1996 to 0.09964 | ns | 0.9882 |
| Negative vs. L-Arginine | -0.1396 to 0.1596 | ns | 0.9997 |
| Negative vs. L-Asparagine | -0.2096 to 0.08964 | ns | 0.9619 |
| Negative vs. L-Aspartic Acid | -0.2096 to 0.08964 | ns | 0.9619 |
| Negative vs. L-Glutamic Acid | -0.2096 to 0.08964 | ns | 0.9619 |
| Negative vs. L-Glutamine | -0.2096 to 0.08964 | ns | 0.9619 |
| Negative vs. L-Histidine | -0.1396 to 0.1596 | ns | 0.9997 |
| Negative vs. L-Isoleucine | -0.1396 to 0.1596 | ns | 0.9997 |
| Negative vs. L-Leucine | -0.1396 to 0.1596 | ns | 0.9997 |
| Negative vs. L-Lysine | -0.1396 to 0.1596 | ns | 0.9997 |
| Negative vs. L-Methionine | -0.1396 to 0.1596 | ns | 0.9997 |
| Negative vs. L-Ornithine | -0.1396 to 0.1596 | ns | 0.9997 |
| Negative vs. L-Phenylalanine | -0.1396 to 0.1596 | ns | 0.9997 |
| Negative vs. L-Proline | -0.1396 to 0.1596 | ns | 0.9997 |
| Negative vs. L-Serine | -0.2096 to 0.08964 | ns | 0.9619 |
| Negative vs. L-Threonine | -0.2096 to 0.08964 | ns | 0.9619 |
| Negative vs. L-Valine | -0.1396 to 0.1596 | ns | 0.9997 |
| Negative vs. Tyramine | -0.2096 to 0.08964 | ns | 0.9619 |
| Negative vs. Glycyl-L-Aspartic Acid | -0.2096 to 0.08964 | ns | 0.9619 |
| Negative vs. Glycyl-LGlutamic Acid | -0.2096 to 0.08964 | ns | 0.9619 |
| Negative vs. Glycyl-L-Proline | -0.2096 to 0.08964 | ns | 0.9619 |
| Negative vs. D,L-Octopamine | -0.01509 to 0.01509 | ns | >0.9999 |
| Negative vs. Sec-Butylamine | -0.005093 to 0.02509 | ns | 0.1982 |
| Negative vs. L-Alaninamide | -0.05954 to 0.07954 | ns | 0.9841 |
| Negative vs. L-Alanyl-Glycine | -0.1195 to 0.01954 | ns | 0.1982 |
| Negative vs. L-Homoserine | -0.05954 to 0.07954 | ns | 0.9841 |
| Negative vs. L-Pyroglutamic Acid | -0.05954 to 0.07954 | ns | 0.9841 |
| Negative vs. D,L-Carnitine | -0.005093 to 0.02509 | ns | 0.1982 |
| Negative vs. Putrescine | -0.01509 to 0.01509 | ns | >0.9999 |
| Negative vs. m-Inositol | -0.1315 to 0.09048 | ns | 0.9992 |
| Negative vs. Mono Methyl Succinate | -0.1025 to 0.1195 | ns | 0.9997 |
| Negative vs. Gelatin | -0.06248 to 0.1595 | ns | 0.9608 |
| Negative vs. 1,2-Propanediol | -0.1305 to 0.09148 | ns | 0.9993 |
| Negative vs. 2,3-Butanediol | -0.09198 to 0.1300 | ns | 0.9993 |
| Negative vs. 3-Hydroxy 2-Butanone | -0.07598 to 0.1460 | ns | 0.9984 |
| Negative vs. Chondroitin Sulfate C | -0.06648 to 0.1555 | ns | 0.983 |
| Negative vs. Amygdalin | -0.6605 to -0.4385 | **** | <0.0001 |
| Negative vs. N-Acetyl-beta-D Mannosamine | -0.1055 to 0.1165 | ns | 0.9998 |
| Negative vs. Acetamide | -0.06348 to 0.1585 | ns | 0.9684 |
| Negative vs. 2-Aminoethanol | -0.07898 to 0.1430 | ns | 0.9986 |
| Negative vs. Phenylethylamine | -0.08898 to 0.1330 | ns | 0.9991 |
| Negative vs. Glycerol | -0.1360 to 0.08598 | ns | 0.999 |
| Negative vs. D-Ribono-1,4-Lactone | -0.06898 to 0.1530 | ns | 0.9855 |
| Negative vs. Dihydroxy Acetone | -0.07548 to 0.1465 | ns | 0.9984 |
| Negative vs. alpha-Cyclodextrin | -0.06698 to 0.1550 | ns | 0.9836 |
| Negative vs. beta-Cyclodextrin | -0.06448 to 0.1575 | ns | 0.9739 |
| Negative vs. gamma-Cyclodextrin | -0.06798 to 0.1540 | ns | 0.9846 |
| Negative vs. Dextrin | -0.1475 to 0.07448 | ns | 0.9983 |
| Negative vs. Glycogen | -0.1290 to 0.09298 | ns | 0.9993 |
| Negative vs. Inulin | -0.06398 to 0.1580 | ns | 0.971 |
| Negative vs. Laminarin | -0.06198 to 0.1600 | ns | 0.9563 |
| Negative vs. Mannan | -0.06248 to 0.1595 | ns | 0.9608 |
| Negative vs. Pectin | -0.1045 to 0.1175 | ns | 0.9998 |
| Negative vs. Tween 20 | -0.1145 to 0.1075 | ns | 0.9999 |
| Negative vs. Tween 40 | -0.1615 to 0.06048 | ns | 0.9413 |
| Negative vs. Tween 80 | -0.1355 to 0.08648 | ns | 0.999 |
| Negative vs. 2-Deoxy Adenosine | -0.1410 to 0.08098 | ns | 0.9987 |
| Negative vs. Adenosine | -0.1625 to 0.05948 | ns | 0.93 |
| Negative vs. Inosine | -0.2255 to -0.003522 | * | 0.038 |
| Negative vs. Thymidine | -0.1220 to 0.09998 | ns | 0.9996 |
| Negative vs. Uridine | -0.1750 to 0.04698 | ns | 0.6989 |
| Negative vs. alpha-Methyl-D-Galactoside | -0.1215 to 0.1005 | ns | 0.9996 |
| Negative vs. alpha-Methyl-DMannoside | -0.06248 to 0.1595 | ns | 0.9608 |
| Negative vs. a-Methyl-D Glucoside | -0.07898 to 0.1430 | ns | 0.9986 |
| Negative vs. beta-Methyl-D Glucoside | -0.1150 to 0.1070 | ns | 0.9999 |
| Negative vs. beta-Methyl-DGalactoside | -0.07298 to 0.1490 | ns | 0.9982 |
| Negative vs. beta-Methyl-DXyloside | -0.06148 to 0.1605 | ns | 0.9514 |
| Negative vs. Arbutin | -0.4595 to -0.2375 | **** | <0.0001 |
| Negative vs. Salicin | -0.4375 to -0.2155 | **** | <0.0001 |
